# Computer Vision-Based Steel Surface Defect Detection: A Survey

**DOI:** 10.3390/s26216705

**Published:** 2026-10-23

**Authors:** Xiang Zou, Yahan Yao, Jiaqing Chen, Chengjun Xu, Tao Liu

**Affiliations:** Jiangxi Provincial Key Laboratory of Intelligent Information Processing and Affective Computing, School of Artificial Intelligence, Jiangxi Normal University, Nanchang 330022, China; 202541600139@jxnu.edu.cn (Y.Y.); 202441600135@jxnu.edu.cn (J.C.); 005627@jxnu.edu.cn (C.X.); liu_tao@jxnu.edu.cn (T.L.)

**Keywords:** steel surface defect detection, deep learning, computer vision, convolutional neural network, YOLO, Transformer, Mamba, industrial quality inspection

## Abstract

Steel surface defect detection is a core component of quality control in steel manufacturing and is crucial for product performance and production efficiency. In recent years, computer-vision-based deep learning methods have decisively surpassed traditional machine-vision approaches built on handcrafted features, propelling steel defect detection into the intelligent era. The review itself is limited to deep learning methods; handcrafted machine vision is background only. This paper provides a comprehensive survey of deep-learning-based steel surface defect detection, citing 184 publications, of which 183 were published between 2015 and 2026. We organize the literature around the three core tasks—classification, detection, and segmentation—and review each task from the perspective of network architecture optimization. We further summarize more than ten public datasets, including NEU and GC10-DET, together with the commonly used evaluation metrics and open-source code resources, and compile literature-reported results for dozens of models, including the YOLO series, DETR, and Swin Transformer. Those figures are copied from the original papers and are not a unified benchmark. Finally, we analyze the core challenges—data scarcity, small-target detection, model generalization, and real-time performance—and discuss future research directions, including high-resolution image processing, few-shot learning, and large-model-assisted quality assessment.

## 1. Introduction

Steel is a foundational material of modern industry, and its surface quality directly affects the performance and service life of downstream high-end products such as automobiles, home appliances, and aerospace equipment. Surface defects not only degrade the mechanical properties and corrosion resistance of products but can also cause severe economic losses and safety hazards. Efficient and accurate steel surface defect detection has therefore become an indispensable component of intelligent manufacturing and quality-control systems.

**Figure 1:**
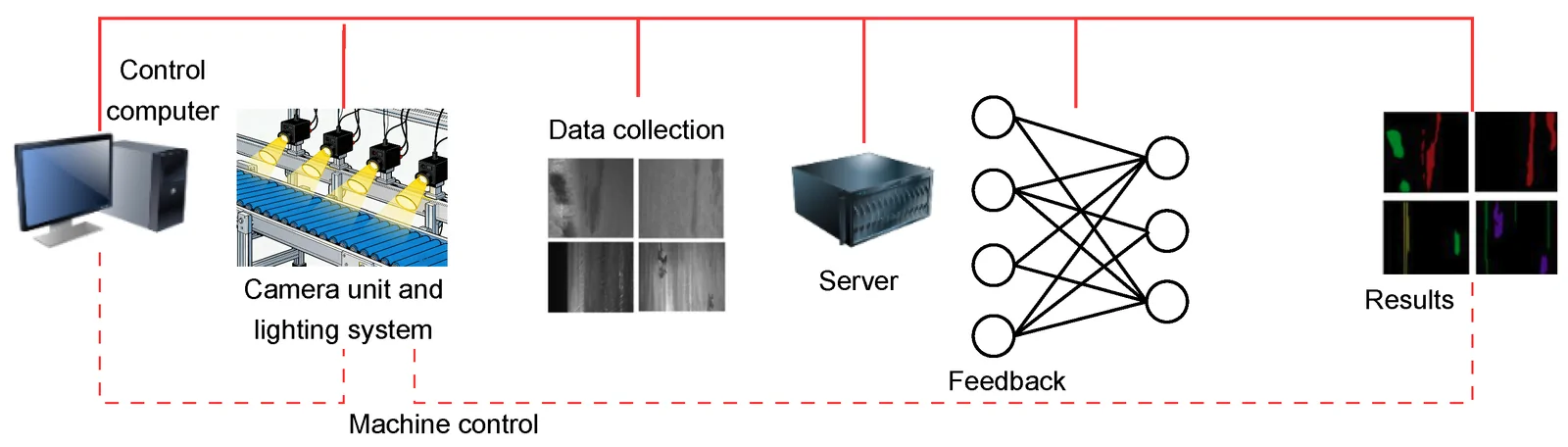
Flow chart of defect detection system.

A typical automated industrial inspection system, as shown in Figure 1, consists of an imaging unit (industrial cameras and specialized light sources), control and acquisition computers, data transmission and server-side processing modules, and feedback and machine-control modules. The workflow proceeds as follows: the imaging unit captures steel strip surface images in real time on the production line; the control computer performs preliminary preprocessing; the server carries out intelligent defect recognition and result analysis; and the detection results are finally fed back to the production control system in real time, enabling quality monitoring and closed-loop control from perception through decision to execution.

Traditional defect inspection relies on manual visual examination or machine-vision methods built on handcrafted features, which struggle to keep pace with high-speed production lines and show clear limitations in efficiency, robustness, and adaptability to complex industrial backgrounds. With the rapid development of computer vision, and of deep learning in particular, steel surface defect detection has undergone a fundamental shift from handcrafted-feature image processing to data-driven intelligent methods. Since 2015, CNN, Transformer, YOLO, and DETR models have been successively introduced into this field, significantly improving detection accuracy and efficiency.

**Figure 2:**
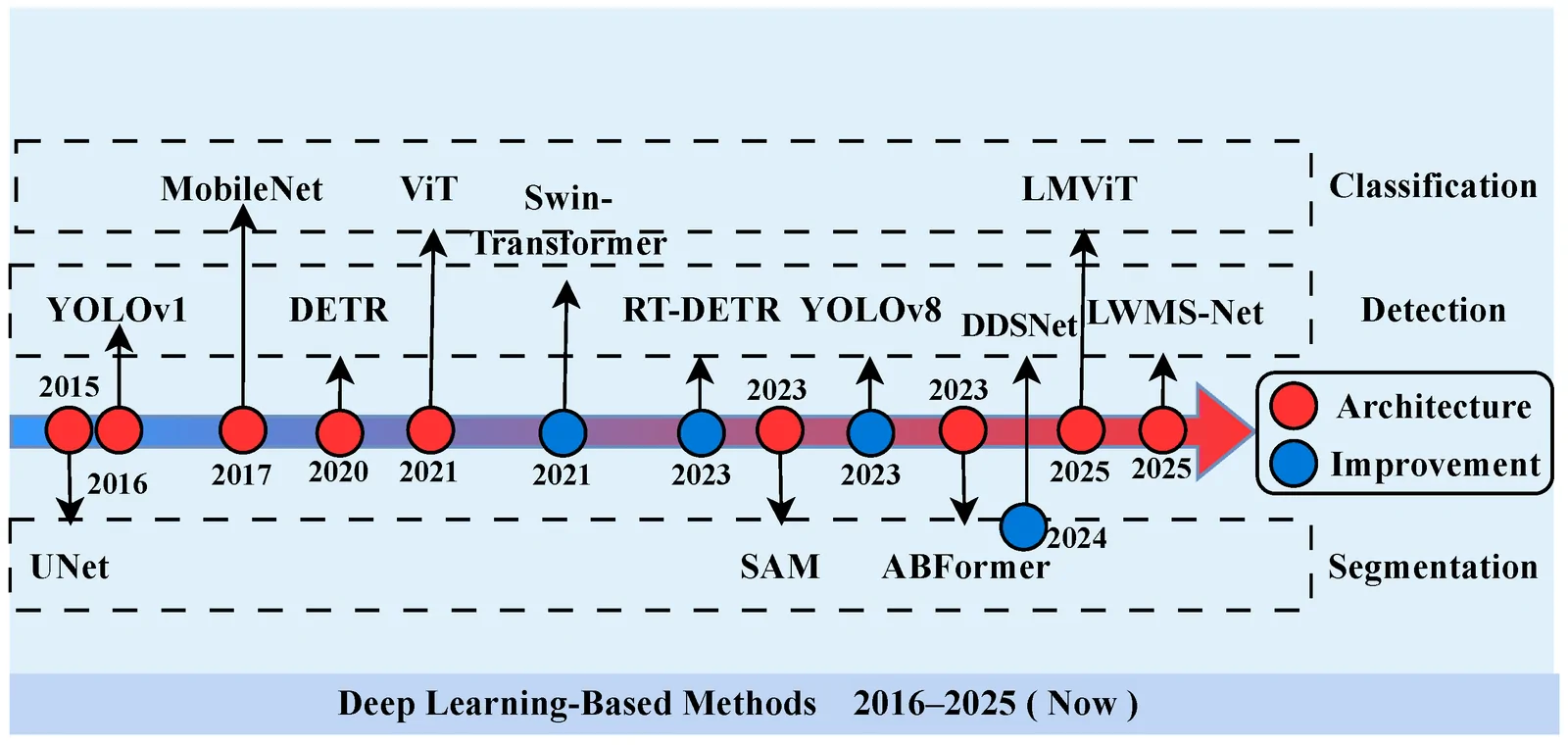
Timeline of steel surface defect detection.

As shown in Figure 2, we briefly outline the development history of deep learning models. Ronneberger et al. [1] proposed the U-Net architecture in 2015 for biomedical image segmentation. Redmon et al. [2] proposed YOLOv1, laying the foundation for single-stage object detection. Google’s research team [3] introduced MobileNets, bringing deep learning to resource-constrained terminal devices. Carion et al. [4] proposed DETR, a new end-to-end object detection framework, in 2020. Dosovitskiy et al. [5] dispensed with convolutions entirely, applying a pure Transformer architecture to image patch sequences for classification. Liu et al. [6] proposed the Swin Transformer, addressing the high computational complexity and lack of hierarchy in ViT. RT-DETR [7], proposed by Baidu’s research team, demonstrated that Transformer-based architectures can also achieve powerful real-time detection. SAM [8], released by Meta AI in 2023, is a breakthrough segmentation model that redefined the boundaries of image segmentation. Ultralytics developed YOLOv8 [9], a cutting-edge object detection model. ABFormer [10] is a Transformer-based boundary-aware fusion framework that handles intra-class differences and inter-class confusion in defect semantic segmentation. Yin et al. [11] proposed DDSNet, a dual-branch network fusing semantic and boundary information. Syed et al. [12] proposed LMViT, which enhances the feature extraction capability of the Vision Transformer for steel surface defect detection by introducing learnable memory modules. LWMS-Net [13], proposed by Zheng et al. in 2025, is specifically designed for steel surface defect detection.

**Figure 3:**
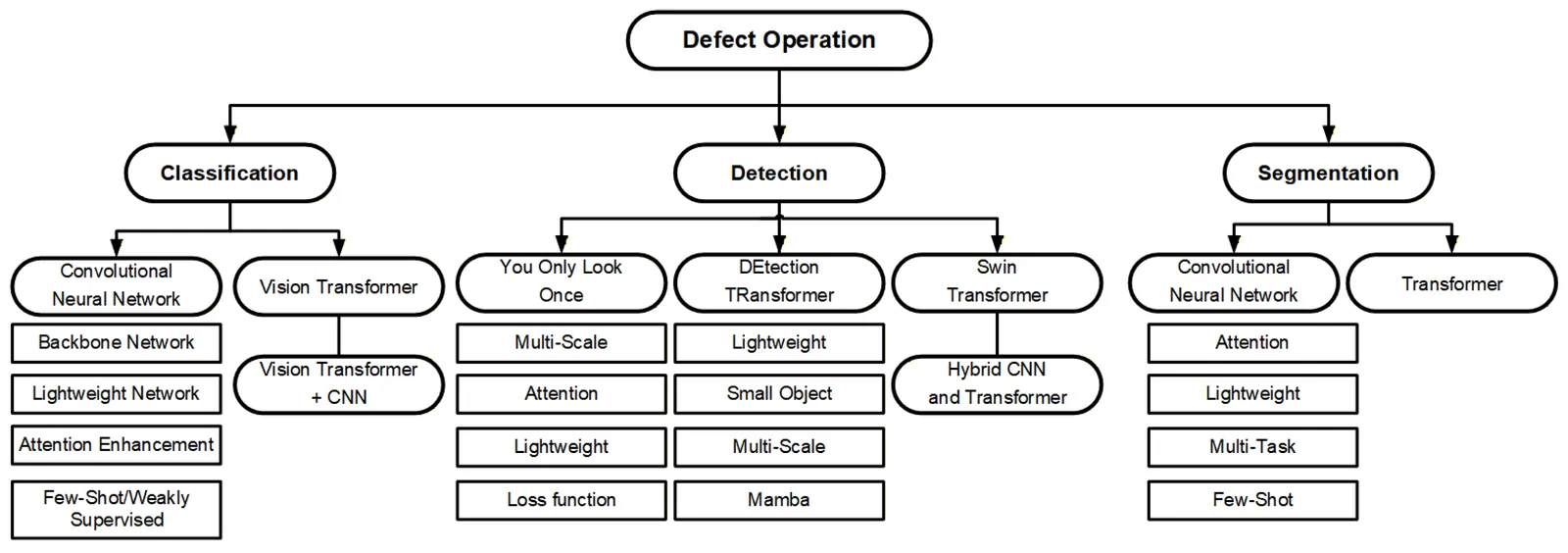
Overall structure of defect operation methods.

Despite these advances, the field still faces substantial challenges at three levels. At the data level: defect sample scarcity, extreme class imbalance, and high annotation costs. At the technical level: insufficient model generalization across production lines and equipment, small targets and complex background interference that degrade detection accuracy, and the tension between real-time performance and accuracy. At the system level: demanding hardware robustness requirements in harsh industrial environments and the complexity of deep integration with existing production systems. In response, researchers have developed a diverse toolbox of solutions, including attention enhancement, weakly and semi-supervised learning, multi-scale feature fusion, and lightweight network design, as summarized in Figure 3, which contrasts the traditional handcrafted-feature paradigm with the deep learning paradigm that has come to dominate this field. This survey systematically organizes computer-vision-based steel surface defect detection methods: it reviews their development, summarizes representative work, analyzes current bottlenecks, and outlines promising future directions, providing researchers and engineering practitioners with a well-structured, comprehensive guide. The methods reviewed in Section 4, Section 5 and Section 6 are deep learning methods. Handcrafted machine vision is used only as background, so the computer-vision title and the deep learning abstract refer to the same scope.

### 1.1. Literature Search Methodology

**Figure 4:**
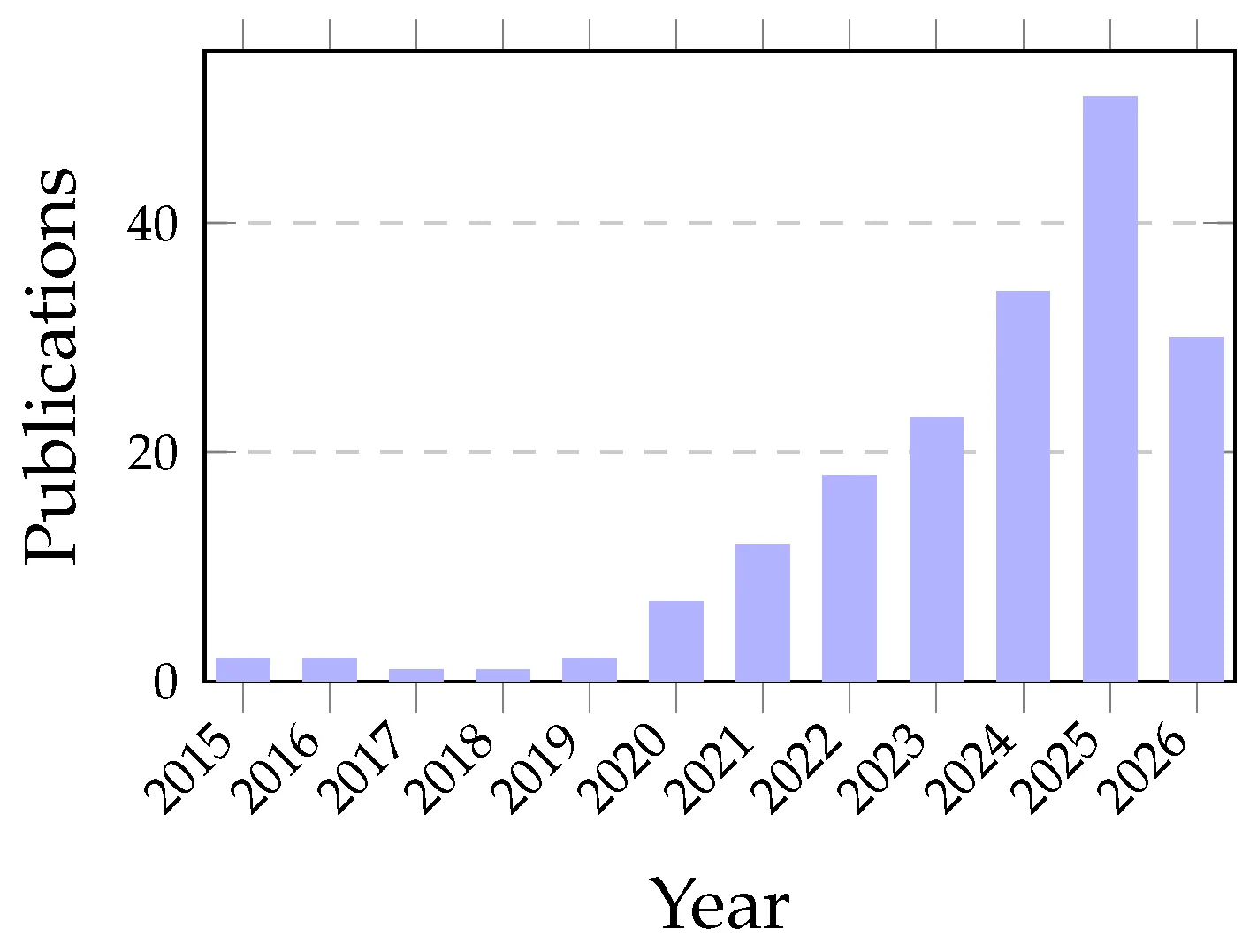
Distribution of the 183 cited publications from 2015 to 2026. One further reference, Du [14], lies outside this window, so the reference list contains 184 items.

**Figure 5:**
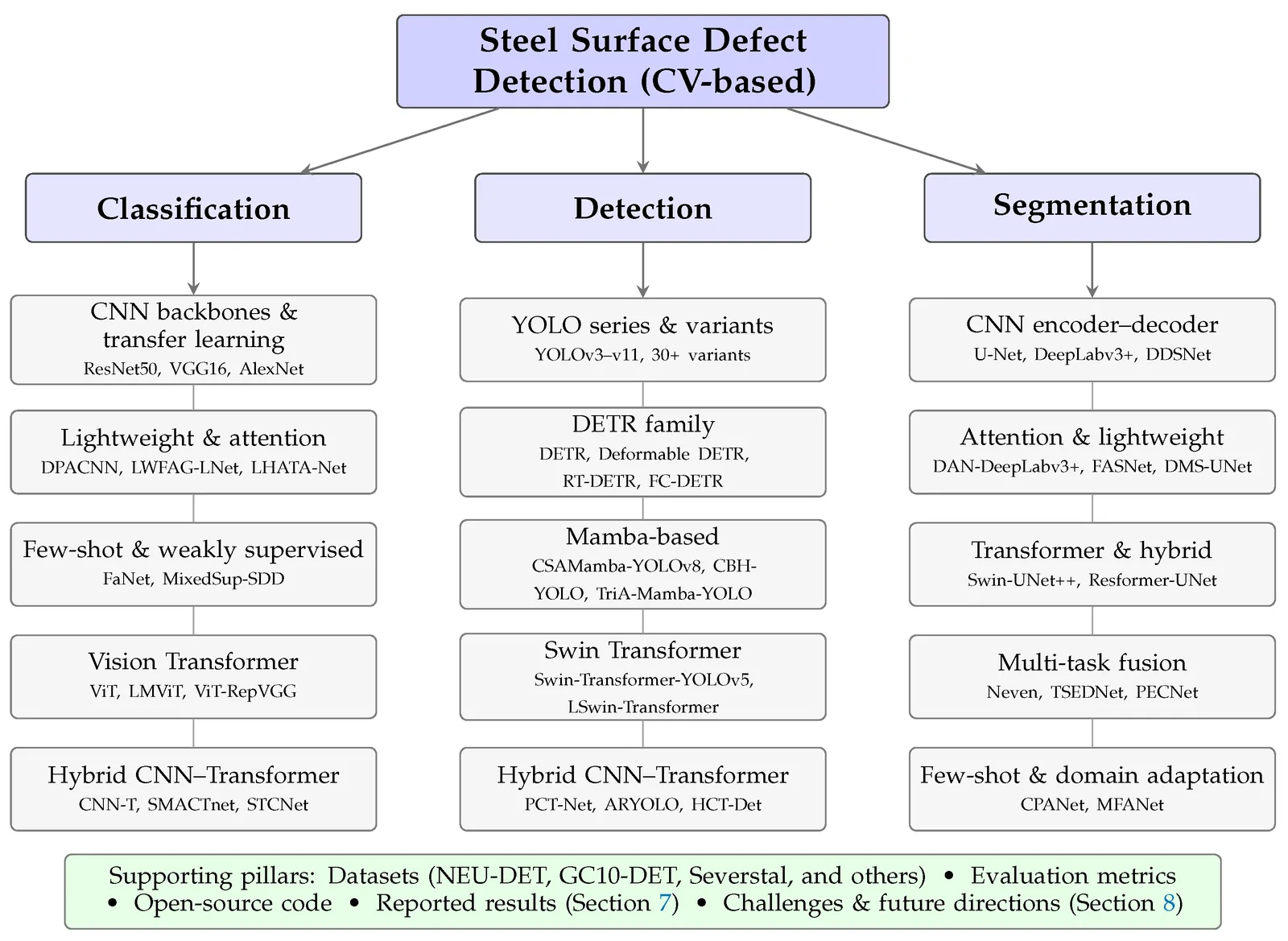
Taxonomy of the surveyed methods, organized by task and architecture family.

To ensure the completeness and representativeness of this survey, we performed a systematic literature search in Web of Science, Scopus, IEEE Xplore, and Google Scholar, covering publications from January 2015 to September 2026. The window opens in January 2015 because that is when the deep learning models later applied to this task, including U-Net and the first YOLO detector, entered the literature cited in Section 1. It closes in September 2026, the month in which the search for this submission was closed. The search queries combined task-related keywords (“steel surface defect detection”, “surface defect classification”, “defect segmentation”), technology-related keywords (“deep learning”, “convolutional neural network”, “YOLO”, “Transformer”, “DETR”, “Mamba”, “few-shot learning”), and domain-specific terms (“hot-rolled strip”, “steel strip”, “surface inspection”). These queries return a large candidate pool. Crossref was not used for screening. One query set on Crossref returns more than 500 records for 2026 alone, and that figure is only an illustration of query size. The records taken into screening came from Web of Science, Scopus, IEEE Xplore, and Google Scholar. Screening then had two stages. In the first stage, titles and abstracts were checked against the following inclusion criteria: (i) peer-reviewed journal or conference papers, with influential preprints retained only for foundational models; (ii) works that target steel surface defect recognition, or a closely related flat-metal surface as defined here; (iii) works that report quantitative results or provide public resources (datasets, code) for reproducible comparison; and (iv) foundational vision methods that are not steel surface papers. In this manuscript those are YOLOv9, Grounding DINO, and SAHI, and they are discussed only as methodological context. Their COCO or other general-dataset results are not treated as steel surface performance. A paper that neither studies an eligible metal surface nor supplies this kind of context is excluded. “Closely related” means a flat aluminum or other rolled-metal surface inspected by imaging, and only when the study also reports results on a public steel dataset or the defect types are the scale, scratch, crack, or inclusion types in Section 2. The aluminum-profile experiments that also use NEU-DET, and the aluminum-strip segmentation study, are included on that basis. ASSM-YOLO is cited for a module used on cathode copper plate defects; that copper-plate result is not entered in Section 7. Papers on unrelated objects are excluded. Publications were excluded when they relied exclusively on non-image-based inspection modalities (e.g., pure ultrasonic, eddy-current, or magnetic-flux approaches without a computer vision component) or lacked verifiable bibliographic metadata. In the second stage, the full texts of the remaining candidates were examined, and the reference lists of existing surveys were snowballed to recover additional relevant works. The reference list contains 184 publications. Of these, 183 were published from 2015 to 2026. Du [14] is cited for the formation of blowholes and falls outside that window. Figure 4 illustrates the distribution of the surveyed publications by year, which clearly reflects the rapidly growing research interest in this field, especially after 2020, whereas Figure 3 sketches the overall paradigm landscape; Figure 5 presents the task–architecture taxonomy under which the selected methods are organized and discussed in Section 4, Section 5 and Section 6.

### 1.2. Related Surveys and Comparison

Several surveys have summarized progress in steel surface defect detection from different perspectives. Table 1 compares the most representative ones with this work. Luo et al. [15] reviewed automated visual defect detection for flat steel products, with particular emphasis on the system-level workflow from imaging to decision making. Wen et al. [16] organized steel surface defect recognition methods primarily along the classification–detection–segmentation task axis. Chazhoor et al. [17] contributed both a review and a benchmark comparison of state-of-the-art methods on public datasets. Sohan et al. [9] focused specifically on the YOLOv8 model and its advancements. The most recent of these entries is Shang et al. [18], a 2026 review of deep-learning-based surface defect detection for strip steel. Table 1 records a full-text check of the five PDFs. “No occurrence” means the named terms do not appear in that file. Luo et al. do not mention NEU-DET, GC10-DET, X-SDD, Mamba, DETR, or YOLOv10–v12; real-time inspection is a main theme, and one cited result is given as 83 FPS. Wen et al. list GC10-DET and X-SDD and tabulate FPS, with FLOPs or parameter counts in some rows; they do not compare one model across those datasets, and domain adaptation appears only as a cited method. Chazhoor et al. run five detectors on NEU with five-fold cross-validation, the highest accuracy being 77.28%; GC10-DET and X-SDD appear only in their related work, and FPS and FLOPs do not occur. Sohan et al. review YOLOv8 in general, with FPS and FLOPs defined for that model; NEU-DET, GC10-DET, and X-SDD do not occur. Shang et al. review strip-steel detectors through 2025, including YOLOv9, YOLOv10, and Vision Mamba. They group published numbers on NEU-DET and GC10-DET and state that comparison across studies is not rigorous; their tables place mAP next to FPS, parameters, or GFLOPs, and they discuss lightweight deployment and domain generalization. What this survey adds is a row-level record of split, input size, pre-training, and augmentation, with every row kept as reference only, plus the rule in Section 1.1 for non-steel papers.

### 1.3. Contributions and Paper Organization

In summary, the main contributions of this paper are as follows:

(1) We provide a unified and up-to-date taxonomy of deep learning methods for steel surface defect detection, organized by the three core tasks (classification, detection, segmentation) and further by architecture family (CNN, Transformer, hybrid CNN–Transformer, Mamba, and YOLO/DETR frameworks), citing 184 publications, 183 of them from 2015 to 2026.
(2) We systematically compile the mainstream public datasets, evaluation metrics, and open-source code resources, offering a practical reference for choosing datasets and metrics, and for method reproduction.
(3) We compile literature-reported results for more than 30 models on NEU-CLS, X-SDD, NEU-DET, GC10-DET, and NEU-Seg, together with the split, input size, pre-training, and augmentation stated by each source. Every row is reference only. The compilation is not a unified benchmark and does not rank architectures.
(4) We analyze the persistent challenges at the data, technical, and system levels and outline promising future directions, including high-resolution processing, few-shot learning, and quality assessment with large models.

The remainder of this paper is organized as follows. Section 2 introduces the main types of steel surface defects and the core challenges of their detection. Section 3 summarizes public datasets, evaluation metrics, and open-source code resources. Section 4, Section 5 and Section 6 review classification, detection, and segmentation methods, respectively, organized by architecture family. Section 7 compiles the literature-reported results and states why they cannot be ranked. Section 8 analyzes future research directions, and Section 9 concludes the paper.

## 2. Overview of Steel Surface Defects and Detection Challenges

### 2.1. Main Types of Steel Surface Defects

#### 2.1.1. External Contact and Mechanical Damage Defects

**Figure 6:**
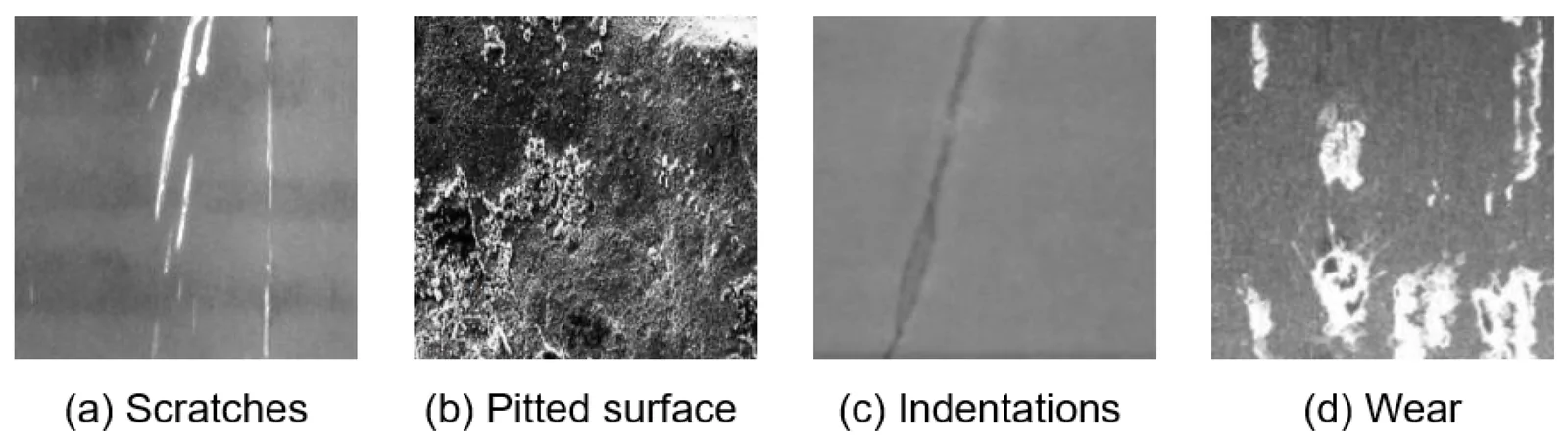
Defect types of external contact and mechanical damage.

In steel production and subsequent processing stages, external contact and mechanical damage are among the main causes of surface defects. This category mainly includes the following types. Scratches [16], usually caused by relative motion between the steel plate and hard objects, appear as continuous or intermittent straight or curved grooves. Pitted surfaces [19], mostly caused by blunt impact or compression, form local depressions. Indentations [16] often originate from protrusions or attachments on the surface of rolling rolls or other equipment and are periodically imprinted on the steel plate during rolling. Finally, wear [20] generally results from continuous sliding friction between the strip steel and equipment, causing gradual loss of surface material. Typical appearances of these defects are shown in Figure 6.

#### 2.1.2. Internal Material and Metallurgical Process Defects

**Figure 7:**
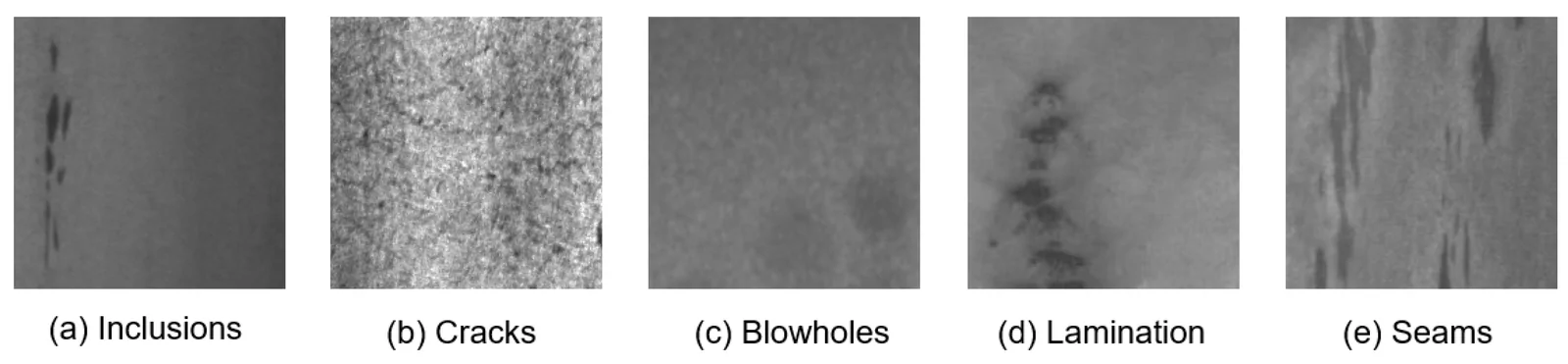
Defect types of internal material and metallurgical process.

During steel manufacturing, internal material and metallurgical processes can also give rise to a series of typical defects. This category mainly includes the following types. Inclusions [16] are non-metallic substances embedded in the steel matrix that appear on the surface as dot-like, strip-like, or irregular heterochromatic spots, mainly originating from foreign matter mixed in during steelmaking. Cracks [21] manifest as linear fractures on the surface, often formed when inherited V-shaped grooves or inclusions in cast billets expand along the rolling direction under rolling force and thermal stress. Blowholes [14] are circular or elliptical holes caused by dissolved gases (such as hydrogen) precipitating during billet solidification. Lamination refers to metal separation layers parallel to the rolling direction, appearing as cracks or peeling. Seams [22] form on the surface of cold-rolled plates through the coupling of multiple factors—material internal inhomogeneity, width-direction deformation stress concentration, and uneven lubrication—resulting in wavy undulation defects. Typical appearances of these defects are shown in Figure 7.

#### 2.1.3. Rolling and Processing Process Defects

**Figure 8:**
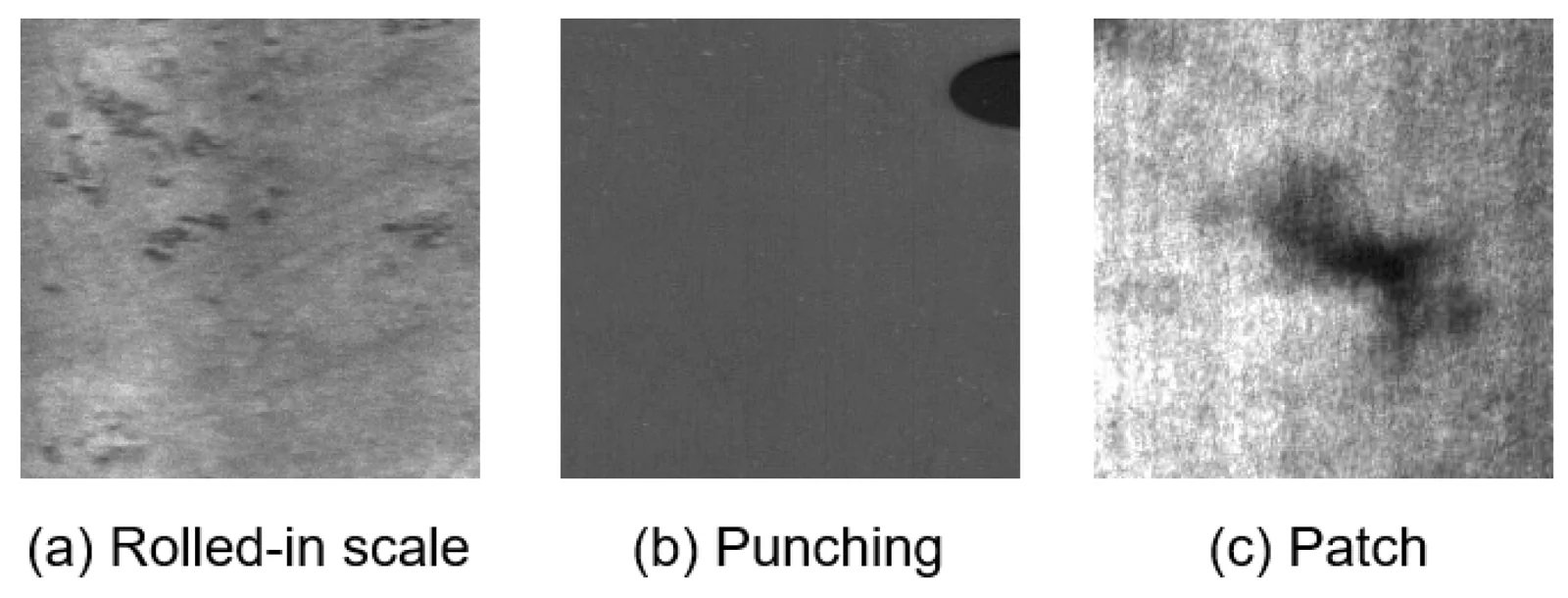
Defect types of rolling and processing processes.

During rolling and subsequent processing, improper process control or operational factors often induce specific types of defects. This category mainly includes the following types. Rolled-in scale [15] appears as irregular black spots or flake-like foreign matter, mainly caused by high-temperature oxide scale that is not completely removed during rolling and is pressed into the strip surface. Patches [23] are surface areas with inconsistent color or texture caused by uneven rolling processes. Typical appearances of these defects are shown in Figure 8.

#### 2.1.4. Chemical Reaction and Environmental Factor Defects

**Figure 9:**
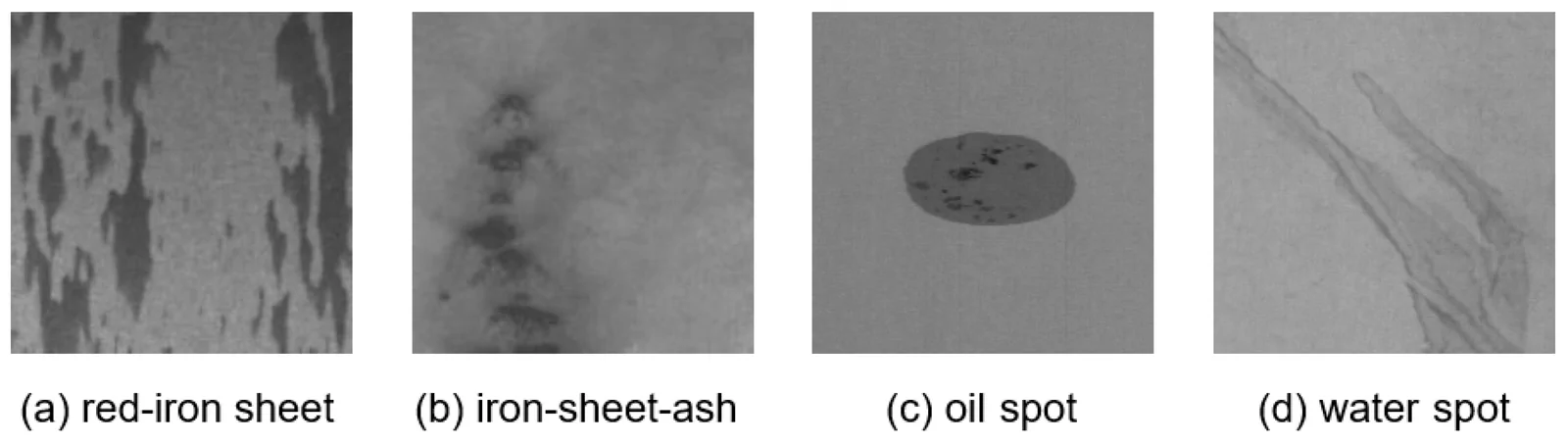
Defects caused by chemical reactions and environmental factors.

During steel production, storage, and transportation, chemical reactions and environmental factors are also important sources of surface defects. This category mainly includes the following types. Red-iron sheet [23] is a red Fe_2_O_3_ oxide layer firmly attached to the surface of hot-rolled high-silicon steel. Iron-sheet-ash [23] is loose, powdered iron oxide scale mixed with dust, forming iron-containing ash. Oil spots [23] are irregular yellow-brown, dark-brown, or iridescent stains formed on the steel plate surface due to incomplete removal of rolling oil or lubricating oil. Water spots [23] are corrosive streaks or imprints caused by contact with water or moisture that was not dried in time. Typical appearances of these defects are shown in Figure 9.

### 2.2. Defect Detection Challenges

#### 2.2.1. Bottlenecks and Challenges at the Data Level

1. **Defect Sample Scarcity and Class Imbalance:** On real production lines, high-quality defect-free steel coils are the norm, while defects are relatively rare, especially severe or rare ones. As a result, defect samples—particularly critical ones—are far outnumbered by normal samples during data collection. This severe class imbalance biases supervised models such as deep convolutional neural networks [24] toward the majority classes during training, producing extremely high miss rates on minority-class defects—precisely the outcome industrial quality inspection seeks to avoid.
2. **Data Annotation Difficulties:** Deep learning is data-driven, and its performance depends on large numbers of high-quality, accurately annotated defect samples. In industry, however, severe defect samples are inherently scarce and annotation requires domain experts, making labeling both costly and slow.
3. **Lack of Unified Defect Definition and Classification Standards:** Neither academia nor industry has converged on a widely accepted, unified defect taxonomy: different steel mills, production lines, and even individual researchers may name, define, and classify the same defect differently. This absence of a universal classification scheme hinders effective knowledge accumulation and horizontal comparison across studies.

#### 2.2.2. Core Technical Bottlenecks

1. **Insufficient Model Generalization Capability and Robustness:** Models trained under specific factory, production line, and steel grade conditions often degrade markedly when transferred to other industrial environments. Lighting conditions, steel plate materials, surface cleanliness, production processes, and defect classification standards all differ across scenarios, which limits model generalization and makes adaptation to new working conditions difficult.
2. **Balance Between Detection Accuracy and Speed:** Traditional machine-vision methods based on handcrafted features and conventional classifiers are fast but struggle with complex and highly variable defect types, leading to insufficient accuracy. Deep learning models can reach substantially higher accuracy, yet their inference cost remains the bottleneck for the millisecond-level response required by high-speed hot-rolled and cold-rolled production lines. Designing “efficient” models that simultaneously satisfy stringent real-time requirements and high accuracy and recall therefore remains a core technical challenge.
3. **Complex Background Interference:** The identification of complex defects faces three main difficulties. First, weak defects such as fine scratches and pitting have extremely low contrast against the background, so their effective features are hard to capture and miss rates are high. Second, defect morphology is highly variable: the same defect type can differ greatly in size, shape, and texture, placing extreme demands on model generalization. Third, background noise formed by oxide scale, texture, and reflections on the steel surface readily generates false defect features while masking real defects, so false alarms and misses coexist.

#### 2.2.3. Challenges in Application Environment and System Integration

1. **Hardware System and Harsh Field Environment Adaptability:** Harsh working conditions on steel production sites—high temperature, high humidity, dust, vibration, and electromagnetic interference—pose severe challenges to the hardware of defect detection systems. Imaging systems must offer high environmental robustness and long-term operational stability, and light source design must suppress strong reflections and deformations on the steel plate surface to obtain high-quality defect images. The technical complexity and implementation difficulty far exceed those of ideal laboratory conditions.
2. **Seamless Integration with Existing Industrial Processes:** Defect detection systems must be deeply integrated into enterprise information architectures, coordinating with manufacturing execution systems, enterprise resource planning, and quality-control systems. The core challenge is to build a bidirectional closed data loop: not only uploading detection results and alarms in real time and automatically generating quality reports, but also linking them with upstream process parameters to support process optimization. Breaking the “information silos” between systems raises cross-disciplinary systems-engineering problems, including industrial software architecture design, communication protocols between heterogeneous systems, and data interface specifications.

## 3. Datasets, Evaluation Metrics and Open-Source Resources

### 3.1. Datasets

Public datasets are the foundation for evaluating and comparing the performance of different detection methods. Currently, multiple standardized datasets targeting steel surface defects are widely used, each with distinct characteristics in defect types, image quantity, resolution, and annotation granularity. This section systematically organizes commonly used datasets applicable to classification, detection, and segmentation tasks, and summarizes their key features to help researchers select appropriate data sources according to task requirements.

#### 3.1.1. Classification Task Datasets

The core of classification tasks is to determine whether defects exist on the steel surface and identify defect categories. The datasets need to cover typical features of different defect types, ensuring that class distribution is consistent with actual industrial scenarios. Table 2 summarizes core information of mainstream classification datasets such as NEU-CLS and X-SSD, including key parameters such as classification objects, sample quantities, and image sizes. These datasets provide standardized support for training and performance validation of classification models, and create conditions for horizontal comparison of different methods.

#### 3.1.2. Detection Task Datasets

Detection tasks not only require identifying defect categories but also need to accurately locate defects in images. Therefore, datasets need to include bounding box annotations for defect regions and consider industrial scenario characteristics such as defect size differences and background complexity. Table 3 organizes core details of commonly used detection datasets such as GC10-DET and Severstal-steel-defect, covering information including detection objects, sample scale, image resolution, and number of defect categories, providing important data support for the development, optimization, and performance comparison of detection models.

#### 3.1.3. Segmentation Task Datasets

Segmentation tasks require pixel-level annotation of defect regions, necessitating precise distinction between defect and background pixels, imposing higher requirements on annotation accuracy and detail completeness of datasets. Table 4 summarizes key information of mainstream segmentation datasets such as NEU-Seg and KolektorSDD2, including segmentation objects, sample quantities, image sizes, and defect categories. These datasets provide high-quality data foundations for training and optimization of segmentation models, and also provide unified benchmarks for performance evaluation of different segmentation methods.

### 3.2. Performance Evaluation Metrics

In steel surface defect detection, model performance is measured along three axes: recognition accuracy (classification), localization precision (detection), and pixel-level consistency (segmentation). This section introduces the metrics commonly reported for each task. Table 5 summarizes the primary and auxiliary metrics per task.

#### 3.2.1. Classification Task Metrics

Classification tasks assign entire images or image patches to specific defect categories. The underlying building blocks are the confusion-matrix elements: true positive (TP), true negative (TN), false positive (FP), and false negative (FN). From these, the standard metrics are derived as follows:

(1) $$\mathrm{Accuracy}=\frac{\mathrm{TP}+\mathrm{TN}}{\mathrm{TP}+\mathrm{TN}+\mathrm{FP}+\mathrm{FN}}$$

(2) $$\mathrm{Precision}=\frac{\mathrm{TP}}{\mathrm{TP}+\mathrm{FP}}$$

(3) $$\mathrm{Recall}=\frac{\mathrm{TP}}{\mathrm{TP}+\mathrm{FN}}$$

(4) $$\mathrm{Specificity}=\frac{\mathrm{TN}}{\mathrm{TN}+\mathrm{FP}}$$

(5) $$F1\text{-}\mathrm{score}=\frac{2\times \mathrm{Precision}\times \mathrm{Recall}}{\mathrm{Precision}+\mathrm{Recall}}$$

Accuracy measures overall classification correctness. Precision reflects the reliability of positive predictions—particularly important when false alarms are costly—whereas Recall measures the completeness of defect recognition, which matters most for safety-critical detection. Specificity measures the model’s ability to exclude non-target samples and is usually reported together with Recall. F1-score, the harmonic mean of Precision and Recall, balances the two and avoids the one-sidedness of a single metric. For multi-class problems, macro- or weighted-averaged variants are typically used.

#### 3.2.2. Detection Task Metrics

Detection tasks additionally require precise localization of defects. IoU measures the overlap between the predicted bounding box $B_{pred}$ and the ground-truth bounding box $B_{gt}$:

(6) $$\mathrm{IoU}=\frac{|B_{pred}\cap B_{gt}|}{|B_{pred}\cup B_{gt}|}$$

IoU ranges in [0,1], and values closer to 1 indicate more accurate localization. Average precision (AP) for a single category is the area under the Precision–Recall curve:

(7) $$\mathrm{AP}=\int_{0}^{1}p(r)\;dr$$

where p(r) denotes precision at recall rate *r*. The mean average precision (mAP) averages ${\mathrm{AP}}_{i}$ over all $N_{c}$ categories:

(8) $$\mathrm{mAP}=\frac{1}{N_{c}}\sum_{i=1}^{N_{c}}{\mathrm{AP}}_{i}$$

The calculation of mAP depends on the IoU threshold: mAP@0.5 fixes the threshold at 0.5, whereas mAP@0.5:0.95 averages over IoU values in [0.5,0.95], following the COCO convention. No universal mAP threshold exists for industrial deployment; values above 80–90% typically indicate strong performance, but detection speed (FPS), model size, and computational cost must be balanced in practice.

#### 3.2.3. Segmentation Task Metrics

Defect segmentation requires pixel-level annotation, so its metrics are computed over the pixel sets of the predicted mask and the ground-truth mask. IoU (the Jaccard index) is the ratio of intersection to union pixels:

(9) $$\mathrm{IoU}=\frac{\mathrm{TP}}{\mathrm{TP}+\mathrm{FP}+\mathrm{FN}}$$

and the mean intersection over union (mIoU), the average over all *k* categories,

(10) $$\mathrm{mIoU}=\frac{1}{k}\sum_{i=1}^{k}\frac{{\mathrm{TP}}_{i}}{{\mathrm{TP}}_{i}+{\mathrm{FP}}_{i}+{\mathrm{FN}}_{i}}$$

is the most widely used comprehensive metric in semantic segmentation. The Dice coefficient,

(11) $$\mathrm{Dice}=\frac{2\mathrm{TP}}{2\mathrm{TP}+\mathrm{FP}+\mathrm{FN}}$$

is more sensitive to small targets and is therefore common in scenarios with tiny defect areas; its values are typically slightly higher than IoU. Pixel Accuracy (PA),

(12) $$\mathrm{PA}=\frac{\mathrm{TP}+\mathrm{TN}}{\mathrm{TP}+\mathrm{TN}+\mathrm{FP}+\mathrm{FN}}$$

is the simplest pixel-level metric, but it can give inflated results when background pixels far outnumber defect pixels, so it is usually reported alongside IoU and Dice as a basic reference. Mean Absolute Error (MAE) and Mean Squared Error (MSE) measure the pixel-wise deviation between predictions and ground truth:

(13) $$\mathrm{MAE}=\frac{1}{n}\sum_{i=1}^{n}\left|y_{i}-{\hat{y}}_{i}\right|$$

(14) $$\mathrm{MSE}=\frac{1}{n}\sum_{i=1}^{n}{\left(y_{i}-{\hat{y}}_{i}\right)}^{2}$$

where $y_{i}$ and ${\hat{y}}_{i}$ denote the true and predicted values at pixel *i*, and *n* is the number of pixels. MAE is suitable when per-pixel errors contribute relatively uniformly, whereas MSE amplifies large errors and is therefore preferred for critical regions (e.g., cracks, holes) where severe localization errors must be heavily penalized.

#### 3.2.4. Metric Selection and Comparison

The primary and auxiliary metrics corresponding to different task types are shown in Table 5.

In summary, classification metrics reflect recognition reliability, detection metrics evaluate localization precision and real-time performance, and segmentation metrics measure pixel-level regional consistency; industrial measurement and quality-control scenarios require a joint view of accuracy, speed, and robustness.

### 3.3. Open-Source Code

Open-source code is a core support for promoting the deployment and innovation of steel surface defect detection technology, not only lowering research barriers but also facilitating method reproduction, comparison, and iteration. The following tables systematically organize representative model open-source resources for the three major tasks of classification, detection, and segmentation, covering key information such as model year, backbone network, and core technical keywords, providing convenient references for researchers to quickly obtain practical tools and verify the effectiveness of new methods.

#### 3.3.1. Open Source Code for Classification Task Models

Classification tasks, as the fundamental component of steel surface defect detection, have the core objective of accurately identifying defect categories, providing basic judgment for subsequent detection and segmentation tasks. In recent years, CNN, Vision Transformer, and hybrid architectures have continuously evolved in this task, giving rise to numerous high-performance models. Table 6 systematically organizes representative classification model open-source resources from 2020 to 2025, covering core information such as model year, backbone network, and technical keywords such as transfer learning, attention mechanisms, and few-shot learning, providing direct technical support for researchers to quickly reproduce classical methods, compare different architectures, and develop new models.

#### 3.3.2. Open Source Code for Detection Task Models

Detection tasks require simultaneous achievement of defect category recognition and location positioning, imposing stringent requirements on models for multi-scale feature capture, background anti-interference, and real-time performance. Models such as the YOLO series, DETR, and Swin Transformer have demonstrated significant advantages in this task through optimization strategies including multi-scale fusion, attention enhancement, and lightweight design. Table 7 summarizes open-source information for various detection models, detailing model names, publication years, backbone networks, and core technical keywords such as adaptive fusion, channel attention, and small-target detection optimization, providing comprehensive reference for engineers in model selection and deployment, and for researchers in improving model architectures.

#### 3.3.3. Open Source Code for Segmentation Task Models

Segmentation tasks require pixel-level precise division of defect regions, which is a key prerequisite for defect quantitative analysis, imposing extremely high requirements on models for boundary modeling and fine-grained feature extraction capabilities. CNN encoder–decoder architectures, Transformer global modeling architectures, and hybrid models of both have become mainstream solutions for this task. Table 8 organizes representative segmentation model open-source resources from 2023 to 2025, clarifying model backbone networks and core technical directions such as multi-scale fusion, edge guidance, and few-shot segmentation, providing important code references for researchers exploring pixel-level defect recognition methods and solving small defect segmentation challenges in complex backgrounds.

## 4. Classification

### 4.1. Method Based on Convolutional Neural Network for Classification

Convolutional Neural Networks (CNNs), with their local connectivity, weight sharing, and spatial pooling, have become a mainstream technology in steel surface defect classification. Compared with traditional methods, CNNs offer three significant advantages. First, they extract defect features automatically without manual design, adapting to complex morphologies and multi-scale variations. Second, they are robust to the industrial challenges of background interference, lighting changes, and low contrast. Third, their computational efficiency facilitates hardware acceleration and real-time detection.

Based on existing research, this section organizes the progress of CNNs in steel defect classification along four directions: (1) basic backbone networks, involving the transfer and improvement of classical models; (2) lightweight networks, focusing on parameter compression and efficiency optimization; (3) attention-enhanced networks, using attention mechanisms to strengthen key features; and (4) few-shot/weakly supervised networks, addressing sample scarcity and high annotation costs.

#### 4.1.1. Basic Backbone Network Category

In steel defect classification, industrial defect images with precise annotations are relatively scarce. Backbone networks pre-trained on large datasets are therefore widely adopted for transfer learning to adapt to the small-data regime of this field. This paradigm transfers general features learned from natural images to defect recognition tasks, significantly improving performance and generalization while avoiding overfitting caused by insufficient data. Pre-trained backbones successfully applied in this field include ResNet and VGGNet families.

Konovalenko et al. [26] developed a classification method for rolled-metal surface defects based on the ResNet50 residual network, achieving 96.91% test accuracy for three types of planar defect images on metal surfaces. VSD network [27] uses the pre-trained VGG19 network as a foundation, systematically extracts and evaluates the quality of its multi-layer feature maps, then optimizes the network structure and parameters, ultimately constructing a VSD network specialized for steel defect classification with stronger performance. Ihor Konovalenko et al. [28] constructed a strong classifier based on pre-trained ResNet50 and ResNet152 models through ensemble learning techniques, and systematically optimized hyperparameters such as loss functions and optimizers to adapt it to high-precision recognition tasks for three types of steel surface defects. AlexSVM [29] proposed an intelligent recognition system based on an improved AlexNet convolutional neural network and support vector machine, achieving 99.7% classification accuracy using only 7% of the features extracted from the FC7 fully connected layer. ResSVM-BO [30] uses pre-trained ResNet101 as a feature extractor, inputs the extracted features into a support vector machine (SVM) for classification, and uses Bayesian optimization to tune the SVM hyperparameters. Ibrahim [31] freezes the convolutional layers of the pre-trained VGG16 model and replaces the classification head to adapt it to new classification tasks, achieving transfer learning. ModAlex [32] builds a new feature extractor by freezing the convolutional layers of the pre-trained AlexNet model and replacing the classifier module, retraining only the fully connected layers. The features output by this extractor are then fed into traditional machine learning classifiers such as KNN and decision trees, constructing a high-performance hybrid classification model.

#### 4.1.2. Lightweight Network Category

Traditional CNN models often suffer from large parameter counts, high latency, and difficulty adapting to production sites. Lightweight network design has therefore become a core research direction for balancing detection accuracy and efficiency, aiming to achieve fast and accurate defect classification with a streamlined structure.

Yang Liu et al. designed a lightweight concurrent convolutional neural network (ConCNN) [33] with a simple structure containing only four convolutional blocks and fully connected layers, and adopted a multi-scale feature fusion strategy. DPACNN [34] constructs a lightweight deep parallel attention convolutional neural network that fuses attention modules and parallel multi-scale modules, focusing on important feature information while reducing parameters and computational load. FDRLNet [35] uses CSPNet as the backbone network to reduce computational redundancy, combines PANet to enhance the feature pyramid, and designs scalable network architectures (such as FNm and FNx variants) to achieve a balance between accuracy and speed. Shengping et al. designed a lightweight network LHATA-Net [36] with few parameters and low computational load, integrating fast and efficient layer aggregation networks, hierarchical multi-scale feature enhancement paths, and lightweight task alignment heads, achieving 54.2 FPS real-time inference while ensuring high detection accuracy. LWFAG-LNet [37] introduces a Legendre multi-wavelet (LW) transform and a feature attention guidance (FAG) mechanism to fuse shallow and deep features, thereby designing a lightweight CNN module containing only four convolutional blocks while significantly reducing network depth. MobileSteelNet [38] builds on MobileNetV1 with multi-scale feature fusion and Cross-Interactive Efficient Multi-Scale Attention.

#### 4.1.3. Attention Enhancement

Steel surface defects often have similar morphologies and inconspicuous rare defect features, causing traditional CNNs to be easily affected by background interference and insufficiently capture key defect features, which degrades classification accuracy. Attention mechanisms, by strengthening effective features and suppressing redundant information, have become a core means to improve the targeting and accuracy of defect recognition.

Kursat Demir et al. [39] construct a network model by parallel training of residual structures and attention structures, and, after filtering deep features through the NCA-ReliefF matching index algorithm, use a support vector machine to complete classification. CNN-T [40] sends locally extracted features from the CNN into a Transformer encoder, using its self-attention mechanism to model features globally and capture long-range dependencies and high-level semantic information. EfficientNet-B0-MMAM [41] constructs a hybrid attention module containing channel and spatial dimensions, and embeds it hierarchically into the EfficientNet-B0 network, enabling the model to adaptively focus on key defect features at different levels. Liu et al. [42] compare sequential, parallel, multi-scale, and residual channel–spatial attention topologies under a unified framework. DPACNN [34], already introduced in the lightweight category above, fuses its attention modules with parallel multi-scale branches so that the network adaptively concentrates on defect-relevant features while remaining parameter-efficient. GFAR-GPAM-CNN [43] enhances the model’s perception of defect locations through the GPAM module, enabling it to accurately focus on defect regions in complex metal backgrounds, thereby distinguishing real defects from background interference. SMACTnet [44] combines sparse and multi-head attention mechanisms, uses a Transformer for global feature extraction to enhance defect category features, and minimizes noise and periodic overlap interference. LWFAG-LNet [37], discussed in the lightweight category above, relies on its feature attention guidance (FAG) module as the attention component that preserves key high-resolution details when fusing deep and shallow features. EAF-DenseNet121 [45] introduces a learnable Sobel edge extraction branch at the input and an Entropy–Attention Fusion module that integrates feature maps, edge maps, and entropy maps into a four-dimensional tensor for dual-path attention fusion, achieving strong classification performance with minimal parameter overhead.

#### 4.1.4. Few-Shot/Weakly Supervised Network Category

Defect samples often exhibit few-shot characteristics due to stable production processes and high annotation costs. At the same time, defects have variable morphologies, complex backgrounds, and large scale differences, which further exacerbate generalization difficulties and make it hard to meet actual industrial inspection requirements. Developing CNNs adapted to this scenario has therefore become an important direction in current research.

Dual-Stream CNN [46] pre-trains the classifier on numerous generated defect samples by constructing a dual-stream neural network containing sample generation and classification training. DenseNet-KNN [47] proposes a “feature extraction + feature transformation + nearest neighbor” classification framework. By comparing three types of feature extraction networks—ResNet, MobileNet, and DenseNet—it finds that DenseNet’s Denseblock is better suited to the degradation problems of few-shot scenarios. EfficientNet-RC [19] combines the EfficientNet deep neural network with randomized classifiers, proposing a fast incremental learning scheme for new defect types that can efficiently learn from very few new samples while avoiding catastrophic forgetting. FaNet [48] constructs a feature-aware network that uses feature attention convolution modules to extract comprehensive features of base categories and fuse cross-layer semantic information, while combining online feature enhancement integration modules to optimize image features of support sets and query sets. NSLNet [49] uses ImageNet as a feature extractor and combines neural structure learning for adversarial training in feature space, solving the problems of insufficient annotated data and weak prediction capability under image perturbations in steel surface defect recognition. HE-NSL [50] enhances the model’s generalization capability under limited training data by performing adversarial training in the extracted feature space. MixedSup-SDD [51] proposes a hybrid supervised end-to-end architecture, combining weak supervision and a small number of fully supervised samples for training, achieving defect detection performance comparable to fully supervised methods while significantly reducing annotation costs. He Zhang et al. [52] proposed a forward-reverse rectification network to address prototype shift in few-shot steel strip defect classification by enhancing feature consistency between support and query samples during training, requiring no extra parameters or inference cost. Zhang et al. [53] proposed a semi-supervised strip steel defect classification model coupling CycleGAN-based data augmentation with a ResNet-50 classifier, tackling the scarcity and largely unlabeled nature of industrial defect data while improving the diversity of generated training samples.

In summary, several converging trends emerge from the CNN-based classification literature: (i) transfer learning from ImageNet-pre-trained backbones (ResNet, VGG, AlexNet families) is the default recipe under limited industrial data; (ii) lightweight and attention-enhanced designs are increasingly preferred over raw backbones, since they reconcile accuracy with embedded deployment constraints; and (iii) few-shot and weakly supervised methods shift the paradigm from “more labels” to “smarter labels”. At the same time, the accuracy figures reported in this section originate from different datasets and evaluation splits, which complicates direct numerical comparison—a standardization gap further discussed in Section 8.

Set against the six fields used in Section 7, these classifiers share the objective of naming a defect class when labels are limited. Transfer from an ImageNet backbone is the usual reported benefit in that setting. Where a source states the cost, it is copied in Section 7: LWFAG-LNet’s compact NEU-CLS setting is 4.39 M parameters, 0.91 GFLOPs and 170 FPS; MGJR is 21.14 M and 2.86 GFLOPs; HiAM-Mamba is 21.25 M and 1.81 GFLOPs. Those speeds are not ranked, because the devices are not stated. The same three papers also report X-SDD accuracies. NEU-CLS and X-SDD are different sets, so their accuracies are not pooled. Few-shot and weakly supervised models apply when annotations are scarce. The forward–reverse rectification method reports no added inference cost. These points do not rank the classifiers. Slender or low-contrast defects are not given a separate classification protocol in this section; the papers instead address classes that look alike and classes with few labels, using attention or few-shot learning. Lightweight models are intended to run faster, but only LWFAG-LNet states 170 FPS, and it does not state the device. What is still missing is one split and one device used by every classifier.

### 4.2. Method Based on Vision Transformer

**Figure 10:**
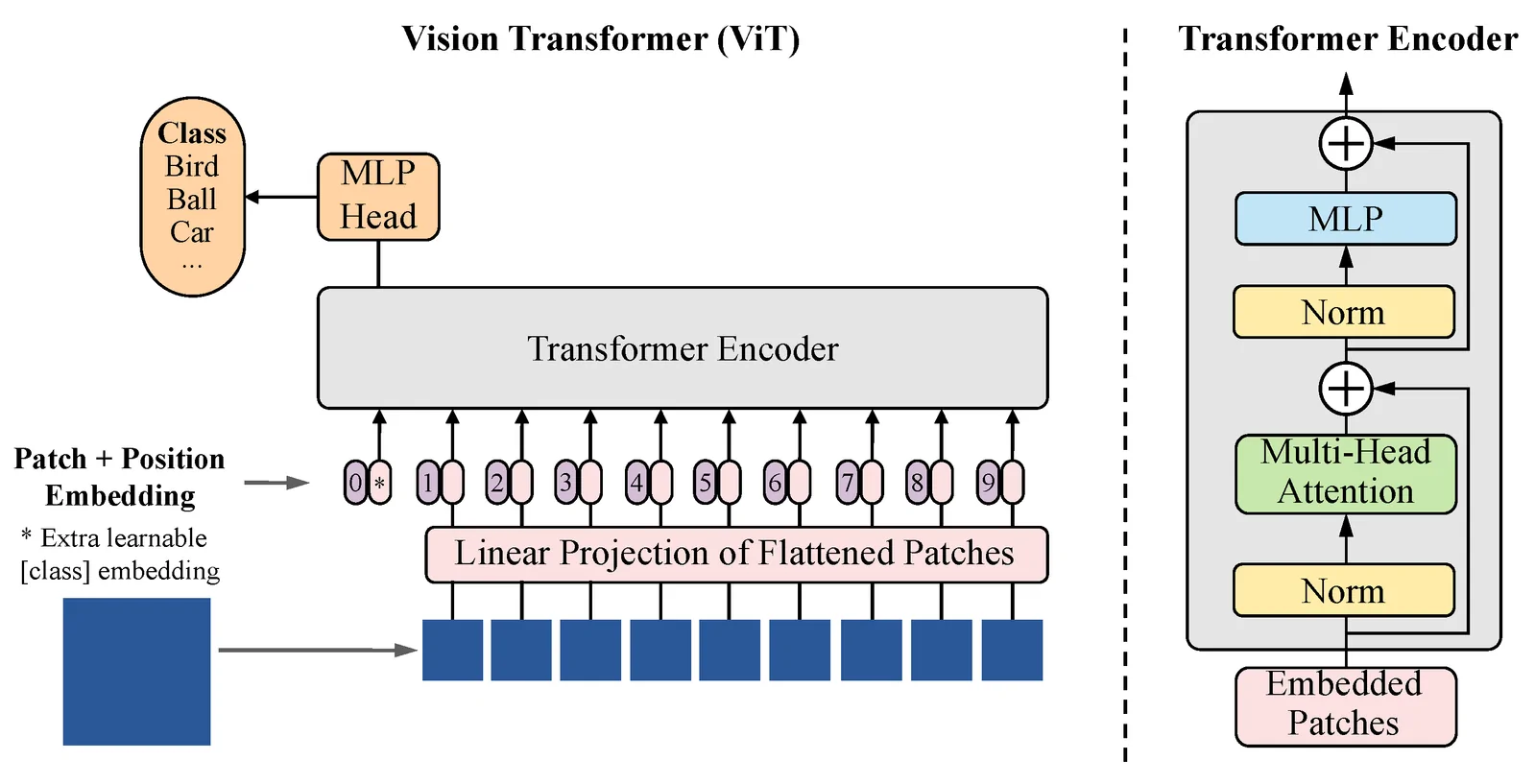
Vision transformer architecture diagram.

ViT (Vision Transformer) applies the Transformer architecture to computer vision tasks. Its core idea is to divide images into patch sequences and perform global modeling through self-attention. Compared with CNNs, ViT offers the following advantages in steel defect detection: (1) self-attention can directly establish global associations between any two image regions, capturing complete defect morphologies and boundary information without stacking many layers; (2) attention weights are generated dynamically from the input rather than being fixed by preset convolution kernels, making the model more adaptable to defects with variable morphologies and random appearances; and (3) global relationship modeling captures the subtle differences between surface defects and normal textures. ViT breaks through the local-convolution limitation of CNNs, extracting global structures and long-range dependencies of defects more efficiently through patch sequence modeling. Vasan et al. [54] achieved high-precision classification of six types of defects on hot-rolled steel surfaces by tuning hyperparameters to find optimal configurations. Spiking-ViT [55] constructed an event-driven hybrid network by replacing the traditional activation functions in ViT with LIF neurons that have more biologically realistic dynamics. LMViT [12] explored the application of a learnable memory Vision Transformer to metal surface defect detection, with data augmentation and t-SNE optimization. Hao Wei et al. [56] introduced RFAConv modules to enhance ViT’s local feature extraction and receptive field expansion capabilities, and combined CBM modules to reduce attention map density and improve noise resistance. Figure 10 shows this architecture.

To fully leverage ViT’s global modeling capability together with CNN’s local feature extraction advantages, hybrid ViT–CNN fusion architectures have become an important research direction in steel defect classification. ViT-RepVGG [57] integrates the ViT self-attention mechanism into stage 3 of the A1-type RepVGG. LSwin Transformer [58] introduces convolutional embedding modules, attention patch merging modules, and deep multi-layer perceptrons, combining CNN’s local feature extraction capability with Swin Transformer’s global dependency modeling. SFN-VIT [59] combines an improved ShuffleNetv2 with integrated SE attention modules as a feature extraction network together with a Vision Transformer, and uses sparse representation techniques for image denoising before input, fusing CNN’s local feature extraction advantages with Transformer’s global modeling capability.

In summary, ViT-based classification methods in this field remain fewer than their CNN counterparts, and their advantages mainly manifest in global context modeling. Pure ViT designs (ViT, Spiking-ViT, LMViT) are still constrained by data requirements, while hybrid ViT–CNN designs (ViT-RepVGG, LSwin Transformer, SFN-VIT) currently offer the most practical compromise between representation power and data efficiency.

## 5. Detection

### 5.1. Method Based on You Only Look Once

**Figure 11:**
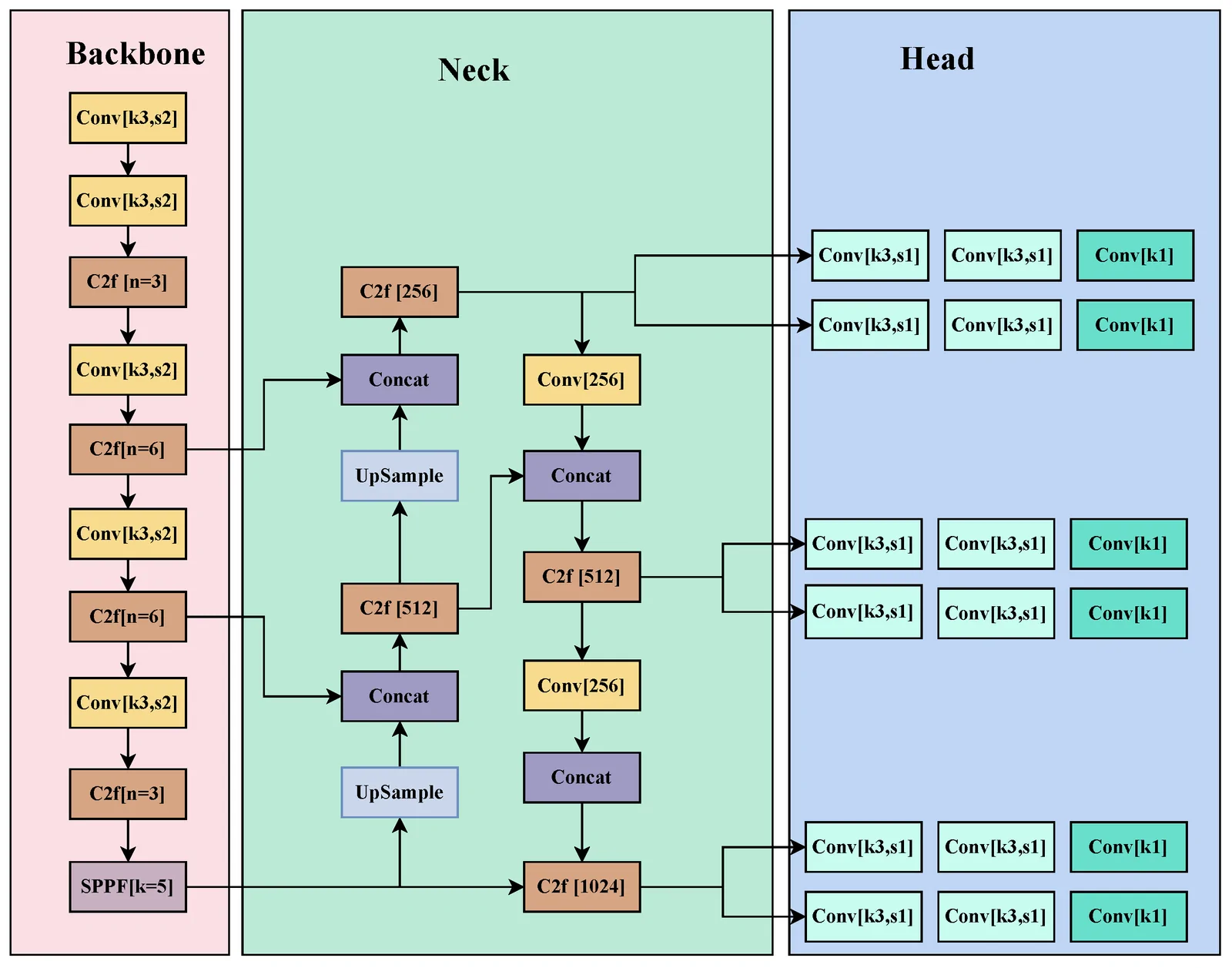
YOLOv 8 architecture diagram.

The YOLO (You Only Look Once) series algorithms demonstrate tremendous application potential in steel surface defect detection with their lightweight design, fast detection speed, and high detection accuracy. Their core advantages include the following: (1) YOLO is convolution-based, with fast training convergence. (2) YOLO performs a single forward pass at runtime, resulting in fast execution. (3) YOLO models have multiple detection heads, providing adaptability to defects of different scales. However, for steel surface defect detection, there are limitations regarding the problems of huge defect size differences, defect class imbalance, and high real-time performance requirements for models. To address this, researchers have optimized YOLO models in the directions of multi-scale feature extraction, attention-based feature fusion, model lightweighting, and loss function optimization. The quantitative results of representative YOLO variants are consolidated in Section 7. Figure 11 shows the YOLOv8 architecture.

YOLOv9 introduces two complementary components: Programmable Gradient Information (PGI) and the Generalized Efficient Layer Aggregation Network (GELAN) [60]. PGI employs auxiliary supervision to improve gradient information during training, while inference uses only the main branch. GELAN generalizes efficient layer aggregation through gradient-path design and flexible computational blocks. These ideas motivate investigation of information preservation and model efficiency in defect inspection, although the original COCO results do not establish their effectiveness on steel surfaces. Domain-specific adaptations have subsequently been investigated; for example, Chen et al. [61] modify YOLOv9 to address small and multi-scale steel defects. Such applications should be assessed using their own datasets, training protocols, and deployment conditions.

Recent studies have adopted YOLO11 as a baseline for surface defect detection. Feng et al. [62] developed YOLO-MFD based on YOLO11n, incorporating multi-scale edge feature enhancement, global-to-local spatial attention-based bidirectional feature fusion, and a grouped detection head. These modifications target subtle-defect representation and model complexity, illustrating how YOLO11 can be adapted to steel inspection. Wang et al. [63] investigated YOLO11-based inspection using NEU-DET and aluminum-profile defects, including deployment on an RK3568 edge device. Together, these studies illustrate adaptations in both network design and deployment. The WSO-YOLO11 and DAQC-YOLO methods discussed below provide further examples of domain-specific modifications. Their reported accuracy and efficiency should be interpreted in relation to each study’s datasets, augmentation strategies, model configuration, quantization, and runtime settings, rather than attributed to the base model alone.

Since many YOLO variants improve several dimensions simultaneously (e.g., a model may combine a multi-scale neck with attention modules and a lightweight backbone), the same model can be discussed under more than one of the following subsections, each time focusing on the corresponding design dimension.

#### 5.1.1. Multi-Scale Feature Extraction and Fusion

Defect sizes on steel surfaces vary greatly, from tiny cracks to large areas of corrosion, which poses enormous challenges for defect detection. Traditional object detection algorithms often struggle to simultaneously handle detection of large-scale and small-scale defects, leading to decreased detection accuracy and recall. To address this challenge, researchers have proposed various improvement strategies to enhance the model’s ability to capture multi-scale features.

YOLOv3 [64] performs detection at three different feature map scales, enabling the model to adapt to defects of different sizes and thereby improving its sensitivity and localization efficiency for multi-scale defects. MSFT-YOLO [65] combines deep semantic information with shallow precise localization information through top-down and lateral connections, thereby generating a series of enhanced feature maps rich in multi-scale contextual information. EFD-YOLOv4 [66] expands the model’s receptive field by introducing a multi-scale module based on hierarchical residual connections. RDD-YOLO [67] extracts multi-scale features by using Res2Net modules in the backbone network and designs a dual feature pyramid network as the neck for deep feature fusion, constructing a richer hierarchical multi-scale feature representation system. CRA-YOLO [68] adopts a multi-scale feature fusion strategy, integrating detail information from shallow network layers with semantic information from deep layers to enrich multi-scale details related to defect targets. CAT-YOLO [69] introduces cross-stage partial FasterNet modules in the backbone network to efficiently extract multi-scale features and designs an adaptive fusion spatial pyramid pooling module to expand the receptive field and optimize cross-level information fusion. Swin Transformer-YOLOv5 [70] adopts the BiFPN structure for multi-scale feature fusion, aggregating feature information from different levels of the backbone network through bidirectional cross-scale connections and weighted feature superposition. MSAF-YOLO [71] performs explicit multi-scale feature extraction by embedding efficient multi-scale convolution (EMSC) modules and uses channel-aware feature mixers to dynamically fuse features under different receptive fields. CBH-YOLO [72] significantly expands receptive field coverage through cross-stage Mamba enhancement modules, combining state space models with large-kernel convolutions. SFMW-YOLO [73] uses space-to-depth convolution to replace traditional downsampling, accurately capturing subtle defect features while preserving spatial resolution. In addition, through multi-scale feature fusion strategies, features from different levels are integrated, further enhancing the model’s ability to capture multi-scale features. MPA-YOLO [74] aggregates multi-level and multi-scale feature information from different depth layers of the network by adding an auxiliary detection head, enabling the model to synergistically utilize shallow detail features and deep semantic features. SFW-YOLO [75] integrates the high-resolution P2 layer from the backbone network into the neck feature pyramid to preserve richer spatial details of small defects; simultaneously adopts a dynamic convolution detection head, adaptively weighing the contributions of different feature layers to multi-scale defects through its scale-aware attention mechanism. WDI-YOLO [76] replaces the standard SPPF module with an SPPELAN module, better capturing multi-scale contextual information through enhanced local attention networks. ASSM-YOLO [77] integrates an adaptive depth feature enhancement module (ADFEM) in the backbone network, which can adaptively adjust network depth and receptive field, thereby capturing complex and variable multi-scale and multi-morphology features of cathode copper plate surface defects. ARYOLO [78] proposes an aggregation-redistribution network that integrates multi-level alignment and fusion redistribution modules to replace the traditional FPN, thereby generating optimized feature maps for YOLO detection heads that fully characterize defects of different scales. FPDNet [79] proposes an efficient deformable layer attention module (CEDLAM), whose core is using deformable convolution to dynamically perceive spatial information, enabling the model to adaptively capture changes in target shape and scale. HDFA-Net [80] achieves collaborative optimization of multi-scale defect features in the frequency domain by introducing a high-dimensional decoupled frequency attention module (HDFA), enabling the network to simultaneously capture large-scale background semantics and subtle defect features. PCT-Net [81] designs an attention-guided local and global feature fusion module in the neck network, achieving deep fusion under local detail enhancement and global semantic association by synergistically utilizing CNN’s local detail features and Transformer’s global contextual information. DAQC-YOLO [82] alleviates information bottlenecks in the backbone network by designing a multi-branch auxiliary supervision framework, while integrating dynamic head modules with detection modules, constructing a DynamicDetect detection head to adaptively fuse features from different levels. OCC-DEIM [83] introduces a geometry-adaptive receptive field mechanism via two modules, CSMPG for morphology adaptation to elongated and irregular defects, and CDCMB for directional sensitivity to rolling defects, achieving 89.4% mAP@0.5 on NEU-DET and 90.4% on GC10-DET. WSO-YOLO11 [84] targets the small, slender, strip-like and low-contrast defects of hot-rolled strips: a wavelet-space-to-depth hybrid stem preserves fine-grained textures during downsampling, and an orientation-strip residual neck strengthens the modeling of elongated structures within the FPN/PAN architecture. Li et al. [85] proposed MCD-YOLO, an improved YOLO method for detecting mixed-class defects on steel plate surfaces.

#### 5.1.2. Attention Mechanisms for YOLO

Under complex industrial backgrounds, noise such as textures and uneven lighting on steel surfaces interferes with model judgment. The introduction of attention mechanisms aims to enable models to focus on key regions in images (i.e., defects themselves) like the human visual system, and suppress weights of irrelevant background information, thereby improving the signal-to-noise ratio of feature representation. In YOLO models, attention mechanisms are used as “plug-and-play” modules to enhance feature representation and fusion efficiency.

MSFT-YOLO [65] introduces TRANS modules based on the Transformer architecture in its backbone network and detection heads, enabling the model to extract and fuse global information. CBAM-YOLOv5 [86] embeds CBAM attention modules in its backbone network, enabling the model to infer attention weights sequentially along two independent dimensions of channel and space, thereby enhancing extraction of key defect features and suppressing irrelevant background information. EFD-YOLOv4 [66] designs a feature alignment module containing attention mechanisms, enabling the network to more accurately align and focus on key positions related to defects when fusing multi-scale features through this module. CRA-YOLO [68] enhances the model’s perception capability of details and global features by introducing CSPLayer Res2Attention (CRA) residual modules, using their internal Res2Attention structure to perform hierarchical attention calculations among multiple feature subsets. MSAF-YOLOv8n [87] designs a complex module containing dynamic coordinate attention (DCA), enabling the network to dynamically capture long-range dependencies of feature maps in channel and spatial position (height, width) dimensions through this mechanism. Swin Transformer-YOLOv5 [70] introduces Swin Transformer in C3 modules to utilize its window self-attention mechanism for handling complex backgrounds and easily confused defects, while adding CoordAttention coordinate attention modules, enabling the network to simultaneously focus on channel relationships and precise positional information. MSAF-YOLO [71] captures cross-dimensional interaction information by integrating Triplet Attention modules together with EMSC modules into C3k2 structures; simultaneously introduces Channel-Aware Feature Mixer (CAFM), using its channel-aware mechanism to dynamically adjust receptive fields and perform weighted multi-scale fusion, enabling the model to simultaneously capture local defects and global texture patterns on steel surfaces. CBH-YOLO [72] expands receptive fields and enhances multi-scale features by introducing adaptive attention mechanisms in CMMC modules to synergize state space models with large-kernel convolutions, and models high-order correlations among features using hypergraph structures and adaptive feature fusion mechanisms in HSGFN modules. SFMW-YOLO [73] solves the problem of feature fragmentation in complex cluttered environments by introducing a multi-scale contextual fusion attention module, using context-guided adaptive mechanisms to hierarchically integrate global and local features. MPA-YOLO [74] designs multi-path convolution attention modules (MPCA) to enhance the model’s sensitivity to defect locations, and introduces partial self-attention modules (PSA) in the backbone network, using them to capture long-range dependencies among features. SFW-YOLO [75] enables the model to adaptively optimize feature responses in three dimensions by introducing dynamic convolution detection heads (DyHead), making them synergistically operate attention mechanisms in scale, space, and task three dimensions. WDI-YOLO [76] enhances feature discriminative power in spatial and channel dimensions by embedding spatial and channel collaborative attention (SCSA) in C2f modules, and introduces triplet attention (Triplet Attention) before detection heads to suppress background interference. ASSM-YOLO [77] integrates SimAM attention mechanisms in the AS feature extraction structure of its backbone network, inferring 3D attention weights for feature maps without additional parameters, thereby adaptively enhancing key defect features and suppressing redundant information. PCT-Net [81] constructs CTBlock modules mixing CNN and Transformer in the backbone network to utilize their self-attention mechanisms to capture global context, designs ALGM modules in the neck to fuse local and global features in an attention manner, and integrates TPGM position guidance modules in detection heads to focus on defect regions, thereby synergistically achieving full-process attention-driven from feature extraction to localization. ARYOLO [78] introduces an attention mechanism in its designed fusion redistribution module (FRM), generating semantically adaptive weights by utilizing global information. CSAMamba-YOLOv8 [88] integrates channel and spatial attention mechanisms into Mamba structures, adaptively calibrating feature responses in channel dimensions and spatial positions. FPDNet [79] proposes CEDLAM modules, combining the adaptive spatial perception capability of deformable convolution with attention mechanisms, enabling the model to dynamically focus on key regions of defect targets. HDFA-Net [80] proposes high-dimensional decoupled frequency attention (HDFA) modules, decoupling features into low-frequency and high-frequency components in the frequency domain, and applying different attention mechanisms to them respectively. AMPNet [89] constructs context and spatial attention (CASA) modules, combining spatial attention (SA) within them with strip convolution, enabling the network to prioritize and focus on key spatial regions where defects are located.

#### 5.1.3. Lightweighting for YOLO

To meet industrial real-time deployment requirements, especially on embedded devices, lightweight model design is crucial. YOLO-LFPD [90] uses the lightweight network FasterNet to replace the original backbone network to accelerate inference, and introduces structurally reparameterized RepVGG modules and pruning techniques. WSS-YOLO [91] introduces lightweight modules such as GSConv and VOV-GSCSP in the neck network. CAT-YOLO [69] adopts cross-stage partial FasterNet blocks as core components and designs streamlined decoupled detection heads to reduce channel parameters. MSAF-YOLOv8n [87] redesigns detection heads using lightweight multi-scale convolution (LMSC) and constructs efficient modules (such as DCA-GSPPFCSPC). Swin Transformer-YOLOv5 [70] adopts the lightweight network GhostNet as the core design, significantly improving detection accuracy while reducing model parameter count, computational complexity, and weight file size by 36.6%, 40.0%, and 34.7%, respectively. MSAF-YOLO [71] uses Space-to-Depth convolution to replace standard convolution to directly reduce the parameter count. SFMW-YOLO [73] replaces detection modules with improved small-target detection heads and introduces feature re-extraction modules based on Space-to-Depth convolution. SFW-YOLO [75] applies pruning techniques to optimize the model framework. WDI-YOLO [76] uses lightweight convolutions such as GSConv to replace standard convolutions in the backbone network, replaces SPPF modules with the more efficient SPPELAN, and directly reduces the output channel count of C2f modules. ASSM-YOLO [77] introduces a Slim Neck structure based on VoV-GSCSP in the neck network, solving the problem of excessive computational complexity. GSR-YOLO [92] designs efficient modules composed of gated blocks and spatial channel reconstruction blocks, replacing the originally computationally intensive C2f modules in YOLOv8. FPDNet [79] adopts HGNetv2 as a lightweight backbone network and combines GhostConv convolution, reducing model parameter count and computational complexity from the source. AMPNet [89] uses RepViTBlocks to replace traditional convolutions in the backbone network and integrates GhostHead composed of GhostConv in detection heads, significantly reducing parameters and computational costs at the fundamental structure and core component levels. The NanoDet-Plus-based model [93] introduces Ghost blocks into the feature pyramid for efficient feature extraction, combines a spatial-channel attention module for multi-scale feature fusion, and adopts a multi-scale detection head, achieving a 1.17 MB model size and 11 FPS on Raspberry Pi 4B. MSR-Det [94] achieves an effective balance between accuracy and efficiency through HTEM for discriminative texture enhancement, FEM for deep-to-shallow semantic guidance, and LLKBM for efficient receptive field expansion.

#### 5.1.4. Loss Function

Loss functions are “guidance signals” in model training, providing direction for model parameter optimization by quantifying the gap between predictions and ground truth. In steel surface defect detection, their core role is to ensure that models can accurately frame defect locations (localization), recognize defect types (classification), and pay special attention to those difficult-to-detect small defects.

**Figure 12:**
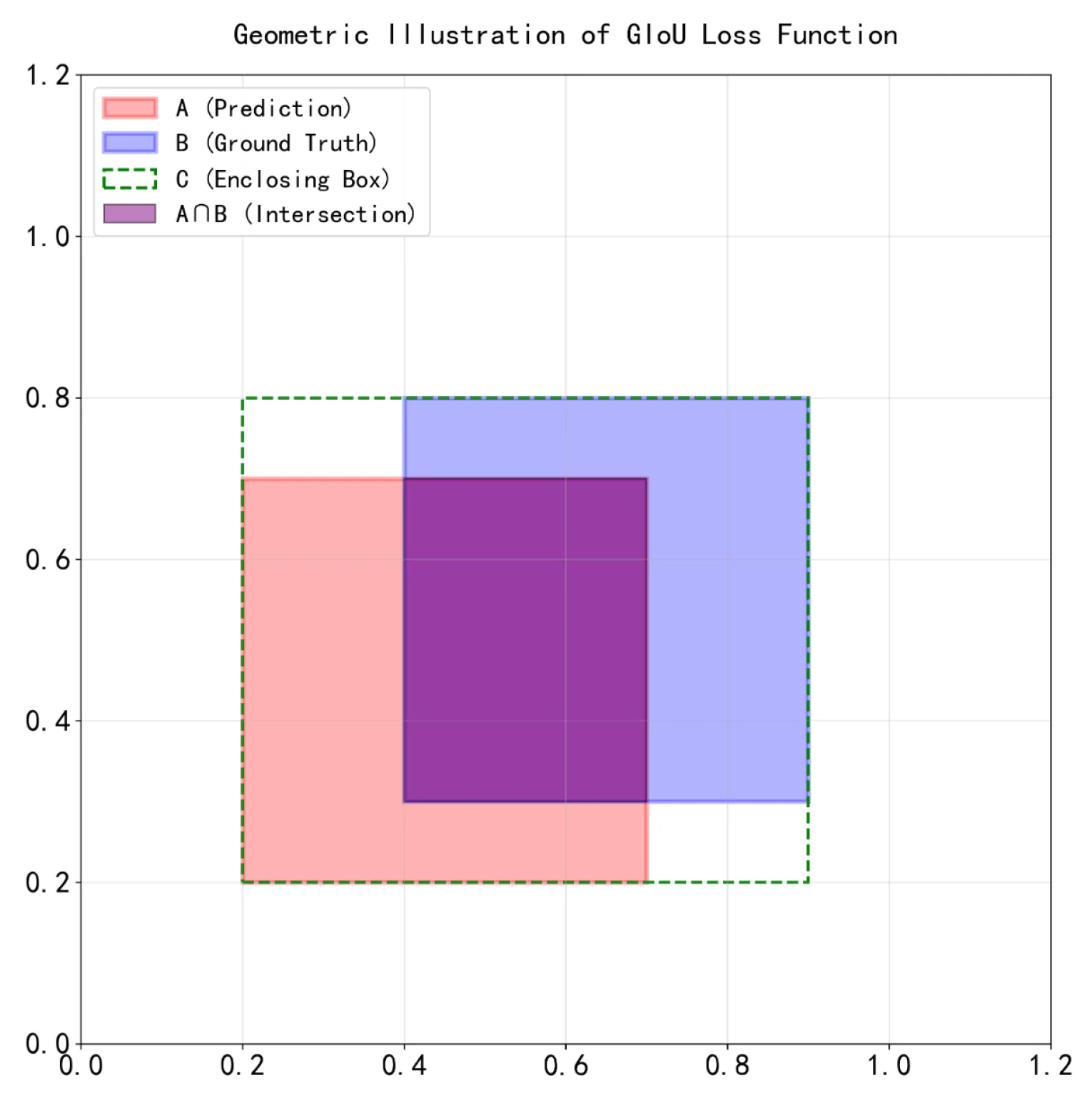
Geometric diagram of GIoU loss function.

In YOLO series models, the evolution of bounding box regression loss functions has undergone a continuous optimization process from IoU to GIoU, CIoU, and EIoU. GIoU solves the gradient vanishing problem of IoU in non-overlapping cases by introducing the minimum bounding rectangle, as shown in Figure 12. Its formula is defined as:

(15) $$L_{GIoU}=1-\mathrm{IoU}+\frac{\left|C-(A\cup B)\right|}{\left|C\right|}$$

where $\mathrm{IoU}=\frac{\left|A\cap B\right|}{\left|A\cup B\right|}$ is the intersection over union, an important metric for evaluating the overlap degree between predicted boxes and ground truth boxes; *A* is the predicted box, *B* is the target box; *C* is the area of the minimum bounding rectangle enclosing predicted box *A* and target box *B*.

**Figure 13:**
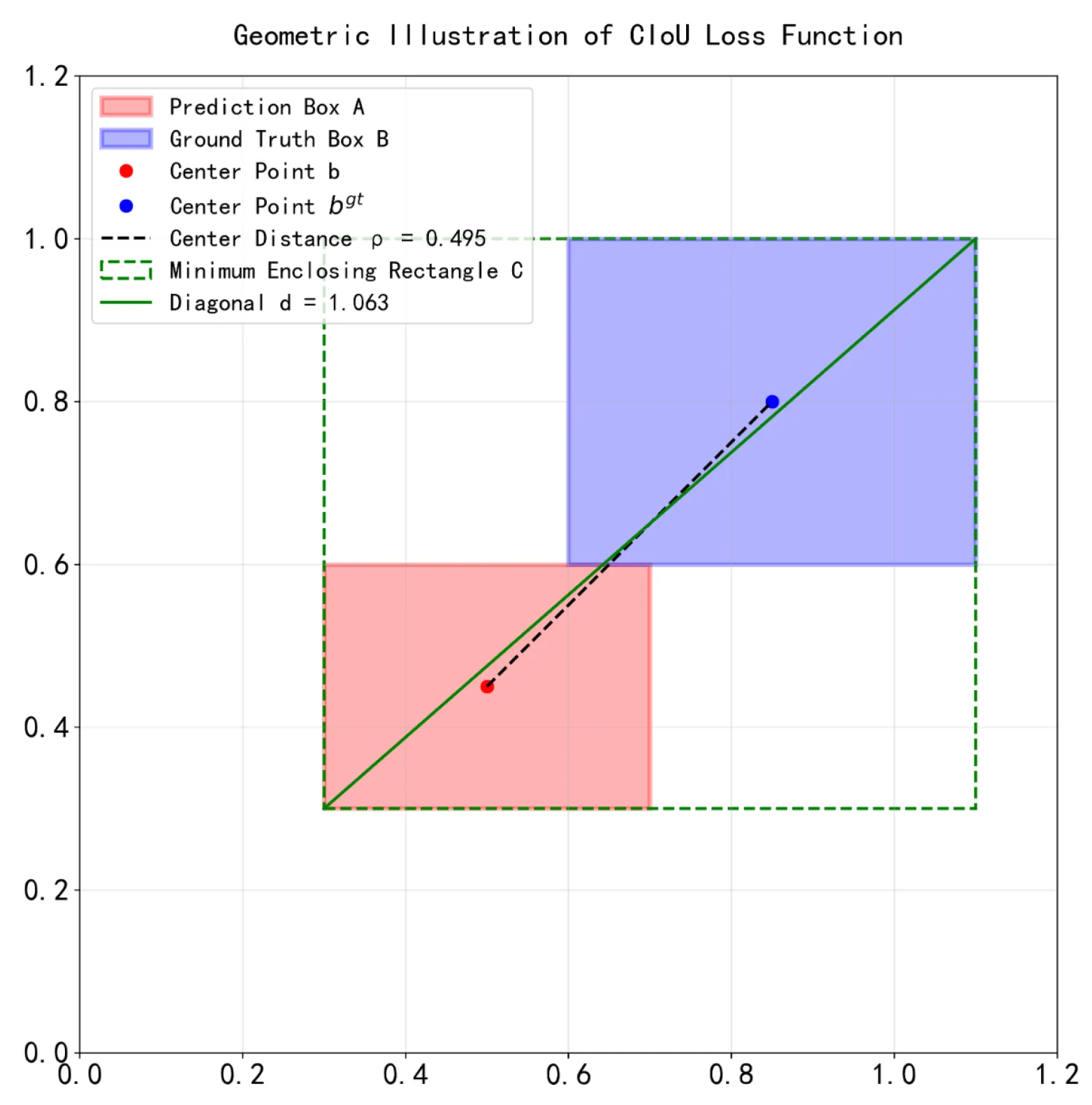
Geometric diagram of CIoU loss function.

**Figure 14:**
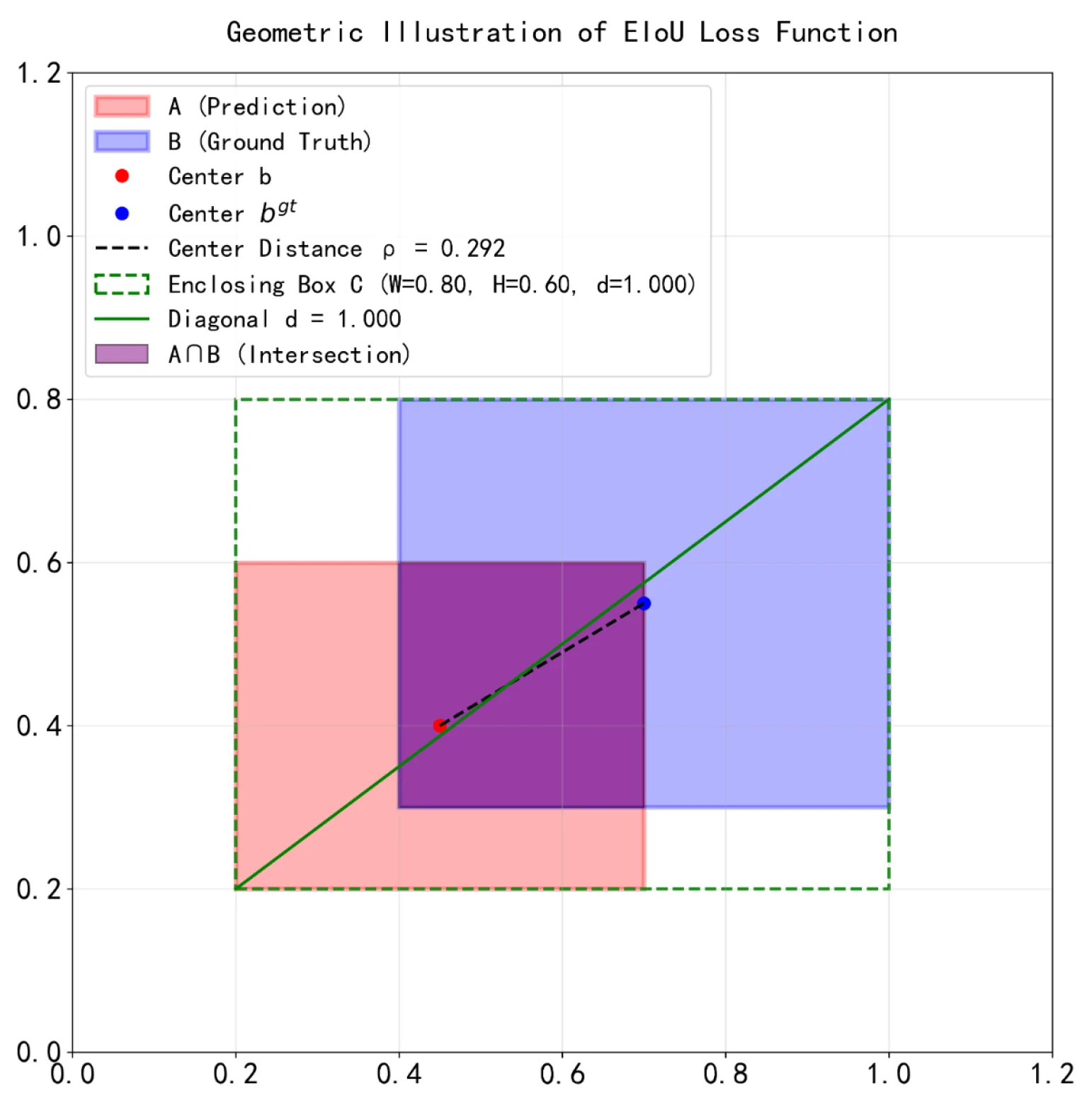
Geometric diagram of EIoU loss function.

CIoU solves the problems of slow convergence and inaccurate regression of GIoU in box containment cases. CIoU believes that a good bounding box regression loss should consider three geometric factors: overlap area, center point distance, and aspect ratio, as shown in Figure 13. Its formula is defined as:

(16) $$L_{CIoU}=1-\mathrm{IoU}+\frac{\rho^{2}(b,b^{gt})}{d^{2}}+\alpha v$$

where $\rho (b,b^{gt})$ is the Euclidean distance between the center point *b* of the predicted box and the center point $b^{gt}$ of the ground truth box; *d* is the diagonal length of the minimum bounding rectangle enclosing the predicted box and ground truth box; *v* is the aspect ratio consistency measure, calculated as: $v=\frac{4}{\pi^{2}}{\left(\arctan\frac{w^{gt}}{h^{gt}}-\arctan\frac{w}{h}\right)}^{2}$; *w* and *h* are the width and height of the predicted box respectively; $w^{gt},h^{gt}$ are the width and height of the ground truth box respectively; α is a trade-off parameter, defined as: $\alpha =\frac{v}{(1-\mathrm{IoU})+v}$;

The EIoU loss function decomposes the aspect ratio loss in CIoU into independent loss terms for width and height, achieving more direct optimization, as shown in Figure 14. Its formula is defined as:

(17) $$\begin{array}{cc} L_{EIoU}= & 1-\mathrm{IoU}+\frac{\rho^{2}(b,b^{gt})}{d^{2}}\\ \; & +\frac{{\left(w-w^{gt}\right)}^{2}}{W^{2}}+\frac{{\left(h-h^{gt}\right)}^{2}}{H^{2}} \end{array}$$

where *W* is the width of the minimum bounding rectangle enclosing the two bounding boxes; *H* is the height of the minimum bounding rectangle enclosing the two bounding boxes.

**Figure 15:**
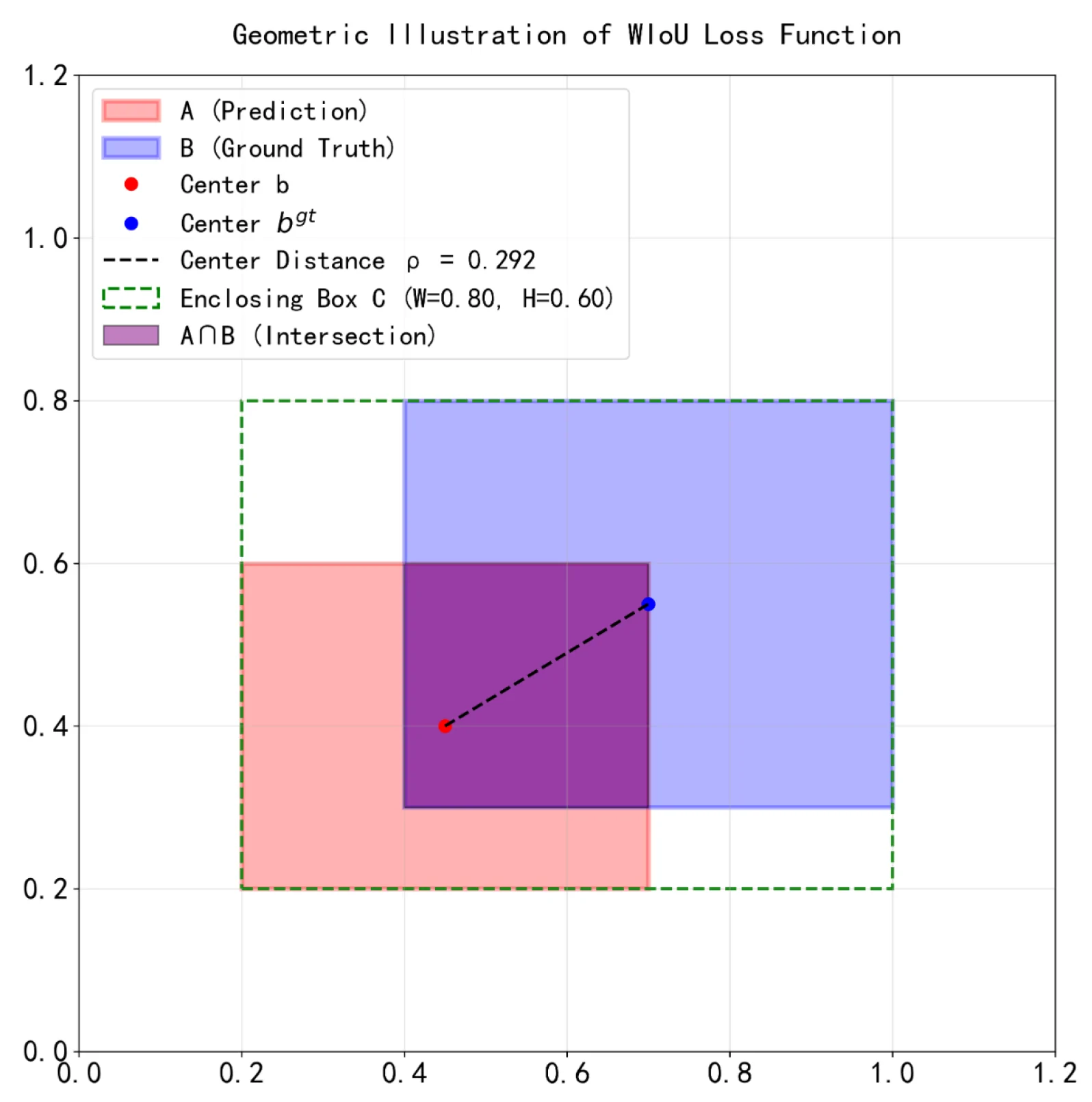
Geometric diagram of WIoU loss function.

Most previous IoU loss functions focus on how to better “define” distance, while WIoU focuses more on dynamically adjusting loss weights, solving the negative impact of low-quality samples (such as blurry, small targets) in training data, as shown in Figure 15. Its formula is defined as:

(18) $$L_{WIoU}=r\cdot R_{WIoU}\cdot L_{IoU}$$

where $r=\frac{\beta }{\delta \alpha^{\beta -\delta }}$, with hyperparameters α and δ; $R_{WIoU}$ is the position weight factor; $\beta =\frac{L_{IoU}^{*}}{\bar{L_{IoU}}}$ is the outlier degree, where $L_{IoU}^{*}$ is the gradient-detached IoU loss and $\bar{L_{IoU}}$ is its running average.

**Figure 16:**
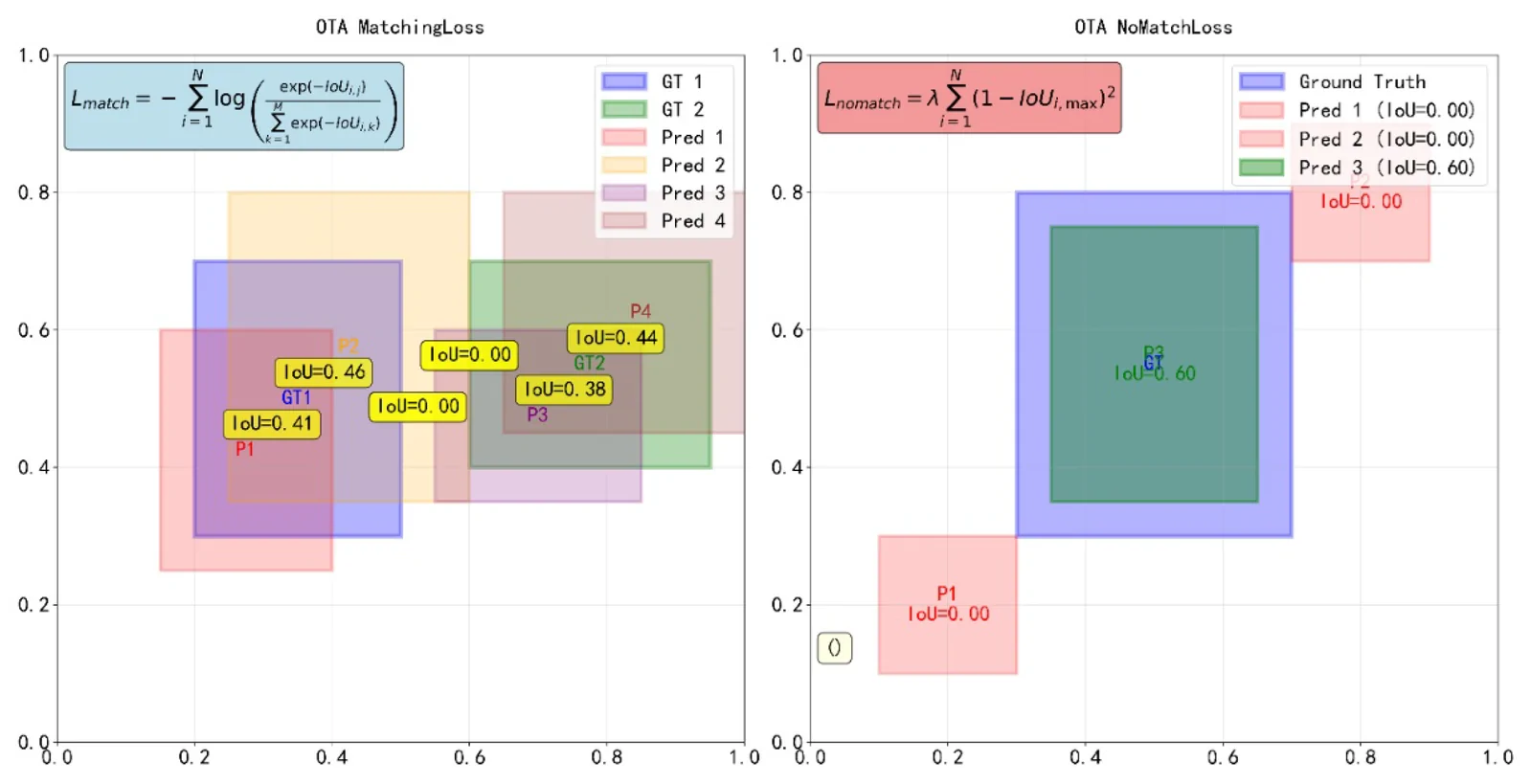
Geometric diagram of optimal transport assignment.

OTA (optimal transport assignment) [95] adopts a dynamic matching mechanism rather than fixed assignment of predicted boxes to ground truth boxes, as shown in Figure 16. OTA formulates label assignment as an optimal transport problem: it seeks the assignment plan that minimizes the total cost of transporting predictions to ground-truth targets, subject to the constraints that each ground-truth box receives exactly its required number of positive samples and each prediction is assigned at most once:

(19) $$\begin{array}{cc} \min_{\pi }\; & \sum_{i=1}^{N}\sum_{j=1}^{M}C_{ij}\pi_{ij}\\ \text{s.t.}\; & \sum_{i=1}^{N}\pi_{ij}=d_{j},\\ \; & \sum_{j=1}^{M}\pi_{ij}\leq 1,\\ \; & \pi_{ij}\geq 0, \end{array}$$

where *N* and *M* are the numbers of predictions and ground-truth boxes, respectively; $C_{ij}$ combines the classification and IoU-based regression costs between prediction *i* and ground-truth box *j*; $\pi_{ij}$ is the transported quantity; and $d_{j}$ is the number of positive samples dynamically assigned to box *j* according to its size, category, and occlusion state.

The optimal transport problem is solved efficiently with a fast Sinkhorn-Knopp iteration, and a dynamic top-*k* strategy further filters out low-quality matches, enabling more accurate assignment of predictions to defects of varying scales and aspect ratios.

For the use of the above loss functions, for example, CBAM-YOLOv5 [86] replaces the bounding box regression loss from GIOU to DIOU based on YOLOv5 to improve localization accuracy and training stability for wear particle detection. YOLO-LFPD [90] uses the OTA loss function to match predicted boxes with ground truth defect boxes through the optimal assignment strategy. WSS-YOLO [91] adopts a WIoU-based loss function and introduces a dynamic non-monotonic focusing mechanism, enabling the model to pay more attention to anchor boxes of ordinary quality during training. SDG-YOLOv5 [96] replaces the original localization loss in YOLOv5’s bounding box regression loss with CIOU Loss and combines it with the Stein divergence loss of the SDG generative model, jointly improving the accuracy and training stability of small-sample imbalanced slab defect recognition. MSAF-YOLOv8n [87] adopts a bounding box loss function mixing NWD and WIoU in a 2:8 ratio, improving sensitivity to small defects and alleviating position sensitivity through NWD, while utilizing WIoU’s dynamic mechanism to optimize training of ordinary quality samples. SFMW-YOLO [73] adopts the Wise-IoU loss function to replace the bounding box regression loss in the original model. SFW-YOLO [75] adopts the EIOU loss function to replace the standard bounding box loss in the original YOLO, directly optimizing differences between predicted boxes and ground truth boxes in center point distance, width-height difference, and overlap area. WDI-YOLO [76] adopts Quality Focal Loss (QFL) to replace traditional loss functions to improve the model’s localization accuracy and generalization capability for weld defects. ASSM-YOLO [77] proposes an MPDSIoU regression loss function that fuses shape matching capability, enhancing bounding box localization accuracy and shape matching capability. GSR-YOLO [92] jointly uses WIoU and SlideLoss to optimize the loss function, utilizing WIoU’s dynamic mechanism to improve training efficiency and generalization capability for ordinary samples, and using SlideLoss to address sample imbalance and easy-hard sample problems. FPDNet [79] adopts the SIoU loss function, redefining penalty metrics by introducing vector angles between required regressions, making bounding box regression more efficient. DAQC-YOLO [82] replaces the original bounding box loss function of YOLOv11s with Unified Intersection over Union (UIoU), which reduces bounding box regression errors by paying more attention to high-quality costs. CFL-YOLO [97] introduces SlideLoss to enhance detection in high bounding-box overlap regions, addressing the sample imbalance and easy-hard sample problems common in steel defect datasets.

Across the four optimization directions reviewed above, a clear convergence can be observed: modern YOLO variants for steel defect detection are increasingly assembled from a shared toolbox—multi-scale fusion modules (e.g., SPPELAN, BiFPN-style necks), plug-and-play attention (CBAM, coordinate attention, triplet attention), slim necks and GhostNet/FasterNet-style lightweight backbones, and WIoU/MPD-family regression losses. This modularization explains the rapid proliferation of YOLO variants in recent years, but it also makes fair attribution of performance gains difficult—an observation that again underlines the need for protocol-aware benchmarking (Section 8).

In those same fields, multi-scale fusion and attention address scale variation and background clutter, lightweight backbones address deployment cost, and the loss variants address localization when classes are imbalanced. A benefit claimed for a module stays inside the study that ablates it. The mAP figures in Section 7 use different splits and input sizes, so they are not used here to name a best YOLO variant. GSR-YOLO is discussed in this section and is grouped with the YOLO rows in that compilation. Slender, low-contrast defects are the case addressed by WSO-YOLO11, which keeps directional structure in the neck. Lightweight backbones are meant to raise speed, but the FPS values in Section 7 were measured on different devices, including an RTX 3090 and an RTX 4060 Ti, so a higher FPS does not by itself mean a faster design. What is still missing is one shared split and one shared device.

### 5.2. Method Based on Detection Transformer

**Figure 17:**
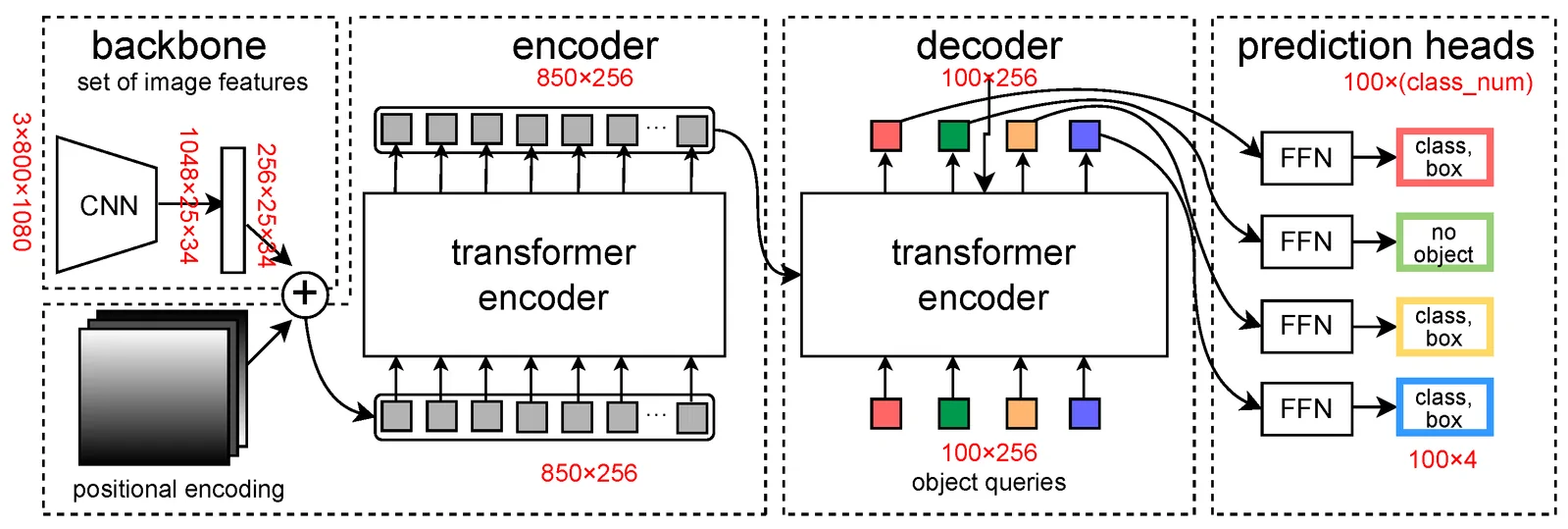
DEtection TRansformer architecture diagram.

DETR (DEtection TRansformer), as a pioneering Transformer-based object detection model, offers a fundamentally different approach from traditional convolutional neural networks for steel surface defect detection. Its core advantages lie in (1) DETR treats object detection as a direct set prediction problem, completely eliminating complex handcrafted components such as anchor generation and non-maximum suppression (NMS), which simplifies the detection pipeline. (2) Its global self-attention mechanism models relationships among all pixels in an image, helping to localize defects in complex backgrounds and understand their contextual information. (3) Its encoder–decoder structure is naturally suited to long-range dependencies, which has potential value for detecting steel surface defects with variable morphologies or low contrast against the background. Figure 17 shows the DETR architecture.

However, DETR models also face a series of challenges in steel surface defect detection. First, their training convergence is usually slower than CNN-based models such as YOLO, requiring higher computational resources. Second, DETR performs poorly on small-target defects because its global attention mechanism may have difficulty precisely localizing tiny defects in high-resolution feature maps. In addition, the initialization and interaction mechanisms of object queries in the Transformer decoder may not be efficient enough for datasets with large defect size differences and class imbalance.

#### 5.2.1. Lightweighting for DETR

The global self-attention mechanism of the original DETR is a computational bottleneck. Deformable DETR substantially reduces computational and memory requirements and accelerates model convergence by introducing deformable attention, allowing each query to focus on only a small number of sampling points on the feature map. LRT-DETR [98] achieves higher detection accuracy and lower computational cost in steel surface defect detection by replacing RT-DETR’s backbone network with MobileNetV3, introducing depthwise separable convolution and VoVGSCSP structures to optimize feature fusion, and adopting the MPDIoU loss function. MESC-DETR [99] significantly improves steel surface defect detection accuracy while maintaining high inference speed (33.8 FPS) by introducing a lightweight Composite-ConvNeXtV2 dual backbone network and edge enhancement modules based on RT-DETR. FTD-DETR [100] significantly reduces computational complexity and memory usage while maintaining high detection accuracy by introducing Faster-EMA modules to replace ResNet18’s basic blocks and adopting deformable attention Transformer encoders.

#### 5.2.2. Small Targets

Deformable DETR [101] uses deformable attention modules to focus on a small number of key sampling points around reference points, while naturally aggregating multi-scale features through multi-scale deformable attention (without relying on FPN), significantly improving detection accuracy for small-sized steel defects while reducing computational complexity and training epochs by 10 times. GM-DETR [102] enhances the ability to distinguish targets from backgrounds by introducing GAM global attention mechanisms, optimizes decoders using layer pruning strategies to reduce parameters, and improves sensitivity to small targets by improving loss functions (such as replacing the L1 loss with MSE). FTD-DETR [100] improves RT-DETR for small target detection in steel defect detection, selecting ResNet18 with low computational complexity and high detection accuracy as the backbone feature extraction network, introducing Faster-EMA modules to improve feature extraction speed and inter-layer interaction, while replacing AIFI modules with Transformer structures with deformable attention encoders, enhancing global perception and multi-scale feature processing capabilities, improving detection accuracy for small targets and other defects while reducing computational complexity.

#### 5.2.3. Multi-Scale

The original DETR faces two major challenges when processing steel surface defects: (1) Multi-scale problem: defect sizes vary extremely (from micron-level cracks to millimeter-level inclusions), with high missed detection rates for small targets; (2) Real-time requirements: industrial production lines need real-time detection speed (>30 FPS). To address these issues, researchers have optimized DETR in the multi-scale direction, mainly focusing on structural improvements, feature fusion mechanisms, and computational efficiency enhancement. RAP-DETR [103] significantly improves detection accuracy and reduces model complexity by introducing multi-scale feature fusion modules CSP-RAB, learnable position encoding, and windmill-shaped convolution PSConv based on RT-DETR. Deformable DETR [101], as discussed above, also provides the core multi-scale deformable attention mechanism that most subsequent RT-DETR-based improvements inherit, efficiently achieving cross-scale feature fusion without relying on FPN. MESC-DETR [99] introduces composite ConvNeXtV2 backbone networks (C-ConvNeXt) and edge-enhanced feature fusion modules (EEFF) based on RT-DETR, significantly improving detection accuracy for small targets and defects with blurred boundaries through multi-scale feature fusion and edge information enhancement. OHEH-RTDETR [104] strengthens extraction of small-defect detail features and fusion of multi-scale features by introducing high-low frequency hierarchical processing frameworks and orthogonal attention mechanisms, thereby achieving DETR model optimization for multi-scale and small-target detection in steel surface defects. ATL-DETR [105] improves RT-DETR for steel surface inspection through a partial-channel directional convolution backbone (APATConv) that models elongated and anisotropic defect patterns via gradient-statistics-based orientation weighting, combined with high-level statistical filtering and local detail fusion. SFC-DETR [106] pursues real-time steel surface defect detection through a lightweight transformer design with adaptive multi-scale feature fusion.

#### 5.2.4. Open-Vocabulary Detection

Grounding DINO extends Transformer-based detection to text-guided open-set detection [107]. It combines a DINO detector with grounded pre-training and integrates visual and language information through a feature enhancer, language-guided query selection, and a cross-modality decoder. This enables object localization using category names or referring expressions. In steel inspection, this capability motivates investigation of text-specified defect categories and annotation assistance. However, its general-purpose benchmark results do not establish reliable zero-shot detection of subtle industrial defects. Domain shift, low visual contrast, defect scale, and prompt formulation therefore require application-specific evaluation. Grounding DINO is included here as methodological context for open-vocabulary inspection, with its potential applications distinguished from validated steel-defect performance.

In summary, DETR-family methods are used where an NMS-free pipeline and global context are wanted. FC-DETR reports 83.60% mAP@0.5 on GC10-DET under its own split (Section 7). That figure belongs only to FC-DETR. It does not show that the DETR family is more accurate or faster than CNN detectors. The lightweight and small-target variants reviewed above change speed and localization inside their own studies. Data requirements and convergence behavior remain open.

### 5.3. Method Based on Mamba

Mamba series models demonstrate excellent performance in long sequence modeling tasks with their advanced State Space Model (SSM) and selective mechanisms, providing new technical paths for steel surface defect detection. Their core advantages include: (1) Mamba models have linear sequence length scaling complexity, capable of efficiently processing high-resolution images and capturing broad contextual information. (2) Their selective SSM core mechanism can dynamically focus on key pixels related to defects, filtering out background noise. (3) The Mamba architecture combines the expressive capability of Transformers with the inference efficiency of RNNs, achieving a good balance between model performance and computational resource consumption. However, when directly applied to dense pixel prediction tasks, Mamba models still have limitations regarding the challenges commonly present in steel surface defect detection, such as variable defect morphologies, weak features of tiny defects, and complex background interference.

#### 5.3.1. Lightweighting for Mamba

Traditional Mamba models and existing vision models have problems of high computational cost and low operational efficiency on resource-constrained industrial detection equipment. Lightweight improvements of Mamba models in steel defect detection can reduce hardware resource requirements while ensuring detection accuracy for precise steel defect identification, adapting to real-time detection requirements in industrial scenarios. CrackMamba [108], although not directly targeting steel, has been successfully applied to crack detection tasks, a field highly related to steel defect detection. This proves the effectiveness of the Mamba architecture in identifying elongated, irregular defect features and provides specific network design and training strategy references. Hu et al. [109] proposed Mamba-HCIM, a lightweight steel plate surface defect detector built on spatial-frequency dual enhancement, further advancing lightweight Mamba designs for industrial deployment.

#### 5.3.2. Attention Mechanisms for Mamba

CSAMamba-YOLOv8 [88] integrates channel attention and spatial attention mechanisms into Mamba. In steel defect detection, by introducing these two attention mechanisms into basic Mamba, the model’s sensitivity to channels containing rich local feature information and attention to key local regions of defects are enhanced, thereby improving defect detection accuracy, robustness, and noise resistance.

#### 5.3.3. YOLO

Leveraging Mamba’s advantages in long sequence modeling to enhance YOLO’s ability to capture complex global contextual information, thereby improving accuracy and robustness in detecting subtle and multi-morphological defects on steel surfaces. CBH-YOLO [72] enhances YOLO’s global context modeling through cross-stage Mamba enhancement modules (CMMC), combining state space models with large-kernel convolutions to significantly expand the receptive field, and further models high-order correlations among features with hypergraph structures and adaptive feature fusion modules. Zhao et al. [110] proposed TriA-Mamba-YOLO, a triple-aware enhancement of the Mamba-YOLO framework for steel defect detection. A discrete wavelet transform preprocessing stage suppresses background noise and strengthens defect feature representation, and the enhancement targets sub-pixel, low-contrast defects as well as extreme variations in defect scale and shape.

In summary, Mamba-based methods are at an early but rapidly growing stage in steel defect detection. Current applications mostly embed Mamba into YOLO-style or CNN frameworks (CBH-YOLO, CSAMamba-YOLOv8), leveraging linear-complexity sequence modeling for global context. Standardized benchmark entries remain scarce, and hardware-aware deployment still needs to be demonstrated at scale.

### 5.4. Method Based on Swin Transformer

Swin Transformer [6], a vision Transformer architecture that introduces hierarchical feature mapping and shifted window mechanisms, provides new solutions for steel surface defect detection. Its core advantages are as follows: (1) the hierarchical structure generates feature maps at different scales, similar to a CNN feature pyramid, making it naturally suitable for defects with large size differences; (2) the shifted window mechanism confines self-attention computation to local windows and achieves cross-window connections through window shifting, reducing computational complexity while maintaining global contextual modeling; and (3) it can be seamlessly integrated as a general backbone into object detection and instance segmentation frameworks such as Mask R-CNN and Cascade Mask R-CNN, providing powerful feature representations for defect localization and segmentation.

However, Swin Transformer also faces several challenges in steel surface defect detection. First, although window attention reduces the computational cost, the parameter count and computational complexity remain relatively high compared with lightweight CNN models. Second, on extremely high-resolution steel surface images, the window division mechanism may disrupt the complete contextual information of some tiny defects, affecting small-defect recognition. In addition, the model requires relatively large amounts of data, making it prone to overfitting when defect samples are scarce. Swin Transformer-BiFPN [111] fine-tunes the Swin Transformer structure to output five-scale features that adapt to defects of different sizes, then fuses features efficiently through a bidirectional feature pyramid network, optimizes sample weights with focal loss, and selects shared detection heads to reduce model parameters. While keeping model size and detection speed basically comparable to classical CNN methods, it improves mean average precision (mAP) by approximately 15.4%. Swin-MFINet [112] proposes a multi-feature fusion network based on Swin Transformer that extracts global semantic and local detail features through Inception encoders, Swin decoders with convolutional layers, and MFI modules, achieving 81.37% and 77.07% mIoU on the MT and MVTec datasets, respectively. Cas-VSwin Transformer [113] proposes a Variant Swin Transformer that optimizes the backbone by strengthening inter-window feature transfer through new window shifting schemes, and constructs a Cas-VSwin Transformer framework that achieves 82.3 box AP on private datasets, outperforming most existing models while validating the superiority of transfer learning for small-scale dataset training. LSwin Transformer [58] achieves 81.2% average precision on steel surface defect datasets by designing convolutional embedding and attention block merging modules, optimizing window shifting strategies, constructing deep multi-layer perceptron modules that fuse CNN and Transformer advantages, and combining transfer learning to accelerate convergence. Swin Transformer-YOLOv5 [70] achieves lightweighting while ensuring accuracy through GhostNet: it introduces Swin Transformer in C3 modules to handle cluttered backgrounds and class confusion, uses CoordAttention modules to strengthen defect feature extraction, and uses BiFPN to optimize feature fusion for scale differences and small-defect detection. The model outperforms mainstream object detection algorithms, improving mAP by 8.39% over the original model while reducing parameters, GFLOPs, and weight size by 36.6%, 40.0%, and 34.7%, respectively, making it more suitable for low-computing platforms.

In summary, Swin Transformer contributes to steel defect detection mainly as a high-capacity backbone and through hybrid designs with CNNs. Its reported gains (e.g., +15.4% mAP for Swin Transformer-BiFPN) come at the cost of additional parameters, making lightweight hybridization (e.g., the GhostNet-based Swin Transformer-YOLOv5) the more practical route for industrial deployment.

### 5.5. Method Based on Hybrid CNN and Transformer

The papers reviewed here combine CNN and Transformer modules so that local texture and longer-range context can be used together. This section does not treat that combination as proof of a higher accuracy. The advantages are threefold: (1) CNN’s powerful local feature extraction precisely captures the texture and edge details of local defects such as tiny cracks and depressions, compensating for the insufficient perception of local fine features in traditional Transformers; (2) Transformer’s global attention mechanism models long-range dependencies between defect regions and the steel surface background, avoiding misjudgments and missed detections caused by background interference, which is especially valuable for defect localization in complex backgrounds; and (3) the papers present the combination as complementary: a CNN keeps local detail and a Transformer adds longer-range context. They do not share one split or one device, so this section does not treat a hybrid as more accurate than a CNN or a Transformer alone.

PCT-Net [81] achieves precise detection of steel surface defects through local and global semantic collaborative modeling by constructing CTBlock feature extraction modules, multi-scale feature fusion modules, and target localization enhancement modules. CRET [114] fuses Transformer’s global contextual modeling and CNN’s local detail perception through class relationship enhancement modules and feature semantic balance bridging modules, enhancing inter-class discrimination while improving detection performance for weak defects, outperforming mainstream methods on multiple industrial defect datasets. MFE-IDD [115] improves detection performance for multi-scale defects—especially low-contrast small holes in weld images—by fusing CNN and Transformer backbone networks with context enhancement and multi-scale feature extraction modules, achieving 93.4% mAP50 and 91.4% average recall while meeting engineering real-time requirements with 57 FPS detection speed. STCNet [116] designs a backbone network (TinystarNet) with star operations, intra-layer interactive encoders, and adaptive cross-scale fusion modules by integrating Transformer and CNN architectures, achieving 92.1% mAP@50 on a self-built SCCB-SDD dataset and striking a balance between accuracy and efficiency with a low parameter count and computational load. HCT-Det [117] enhances local feature continuity while reducing computational complexity through hybrid block structures of window self-attention and residual convolution, and combines multi-scale feature interaction and soft IoU perception mechanisms, achieving 79.5% and 73.3% mAP on the NEU-DET and GC10-DET datasets, respectively.

In summary, hybrid CNN–Transformer designs use a CNN for local texture and a Transformer for longer-range context. The 93.4% mAP50 and 57 FPS reported for MFE-IDD belong to its weld-image experiments, and the 92.1% mAP@50 reported for STCNet belongs to the self-built SCCB-SDD set. These two figures are not one comparison. Low-contrast small holes are the case discussed for MFE-IDD; slender defects are not given a separate protocol in this section. The added fusion modules increase architectural complexity. HCT-Det reports 79.5% mAP on NEU-DET and 73.3% mAP on GC10-DET under its own settings, which still does not rank it against the other hybrids. What is missing is a shared split and a stated device for every speed figure.

## 6. Segmentation

### 6.1. Method Based on Convolutional Neural Network for Segmentation

Convolutional Neural Networks (CNNs), with their powerful local feature extraction capability and translation invariance, have been one of the mainstream methods for steel defect segmentation tasks from early stages to the present. With deepening research, CNN-based segmentation architectures have gradually evolved from basic encoder–decoder structures and differentiated into multiple branches aimed at improving performance, efficiency, and practicality. This section systematically organizes their applications and development in steel defect segmentation around the following core architectural paradigms: (1) attention-enhanced architectures; (2) lightweight optimization architectures; (3) multi-task fusion architectures; (4) few-shot learning architectures. The quantitative results of representative CNN segmentation models are consolidated in Section 7.

#### 6.1.1. Attention-Enhanced Architectures

To enhance the model’s ability to focus on defect regions, attention-enhanced architectures have been introduced. Such methods dynamically recalibrate feature maps by embedding attention modules at key network positions. For example, DAN-DeepLabv3+ [118] integrates dual attention modules for space and channels into DeepLabv3+, enhancing the model’s ability to capture multi-scale contextual information and perform fine-grained segmentation of defect boundaries. DMS-UNet [119] proposes a dual-branch edge extraction module with hybrid attention to enhance context modeling and edge-texture feature extraction, targeting heavy noise interference and low defect–background contrast in steel defect images. Lv et al. [120] combine fused attention mechanisms (FA) with lightweight MobileViTv2 and introduce large-scale feature pyramid networks (LSFPN), improving accuracy and detail perception capability for aluminum strip surface defect segmentation while maintaining high speed. LSA-Net [121] introduces position attention modules (LAM) and shape detection modules (SDM) based on U-Net, enhancing feature differences among different modules through feature difference loss. Zhang et al. [122] introduce multi-scale attention feature fusion modules based on HRNet and combine three targeted training strategies. Li et al. [123] propose a semantic segmentation network that fuses global information (Transformer) with multi-scale local features (MDC-LASPP). DPCSA-Net [124] introduces dual-polarized channel–spatial attention mechanisms and lightweight design in steel defect segmentation. Guclu et al. [125] enhance channel and spatial attention mechanisms by introducing residual squeeze-and-excitation modules (RSB) and residual refinement modules (RRM), and combine synthetic data generation (SDG) to improve the model’s perception and segmentation accuracy for multi-scale defects. Huang et al. [126] introduce fuzzy logic-based feature enhancement modules (FL-FE) and fuzzy loss (FL-Loss), improving the model’s defect segmentation capability under noise interference and foreground-background similarity scenarios. MIAMS [127] introduces multi-image attention modules (MAFE) and main structure feature extraction modules (MSFE). WFF-Net [128] performs weighted fusion of multi-scale features through learnable gating modules and attention mechanisms. Pu et al. [129] enhance feature extraction capability for elongated and tiny defects by introducing geometrically constrained deformable convolutions and spatial correlation perception modules. Zhang et al. [130] achieve dynamic attention allocation and local feature enhancement for multi-scale defects by introducing multi-scale attention modules (MSAA) and efficient local attention modules (ELA), combined with StarNet backbone network.

#### 6.1.2. Lightweight Optimization Architectures

Given the urgent need for real-time detection in industrial production lines, lightweight optimization architectures have become a research hotspot. The core of such methods is seeking the optimal balance between accuracy and efficiency. DSUNet [131] introduces depthwise separable convolution into the U-Net architecture, significantly reducing the model’s computational complexity and parameter count while maintaining high segmentation accuracy. Feng et al. [132] design feature reuse fusion bypasses, lightweight feature refinement modules, and residual atrous spatial pyramid pooling. FASNet [133] proposes suspected defect screening and lightweight feature-aware segmentation networks, achieving high-precision, low-memory real-time segmentation of aviation steel pipe defects on embedded devices through cascaded design and lightweight modules. Rao et al. [134] propose XConv modules and novel Bottleneck modules, decoupling main diagonal and anti-diagonal convolutions and combining axial depthwise convolution. CDARNet [135] introduces cross-dimensional adaptive region reconstruction modules (CDAR) and context self-correlation semantic unification modules (CSSU).

#### 6.1.3. Multi-Task Fusion Architectures

Multi-task fusion architectures improve overall performance by jointly learning related tasks and leveraging task complementarity. For example, Neven et al. [136] construct a dual-branch U-Net structure that simultaneously completes two tasks, defect type segmentation and severity estimation, and introduces production process parameters as additional inputs. TSEDNet [137] introduces task-specific encoder–multi-decoder structures, combining metric learning and domain knowledge, tackling class imbalance and feature information loss problems in steel surface defect segmentation. PECNet [138] proposes a two-stage detection strategy, achieving efficient and precise seamless steel pipe inner surface defect detection through defect rapid filtering and parallel encoder fusion networks.

#### 6.1.4. Few-Shot Learning Architectures

Few-shot learning architectures aim to solve the problems of high annotation costs for steel defect data and scarcity of rare defect samples. Such methods typically follow meta-learning paradigms, performing meta-training on base class datasets containing multiple defect types, enabling models to acquire the ability to quickly learn from few samples. CPANet [23] aggregates long-range associations and multi-scale features of discrete defects under sample scarcity through cross-position proxy modules and spatial compression attention mechanisms. Chi et al. [139] introduce multi-objective domain adaptation and hierarchical dynamic instance segmentation methods, achieving effective transfer and segmentation of steel defect features across multiple source and target domains under scarce labeled samples. Yan et al. [140] propose an uncertainty-driven unsupervised domain adaptation framework, combining local alignment strategies and uncertainty-aware self-training. Shan et al. [141] achieve high-precision segmentation of unseen defect categories with only one annotated sample by introducing bilateral guidance mechanisms and multi-scale receptive field encoders. MFANet [142] proposes a cross-granularity few-shot defect segmentation paradigm and multi-feature aggregation network, training models using coarse-grained annotated data. Hu et al. [143] perform joint training using only a small number of pixel-level annotations and a large number of image-level annotations through multi-stage attention-enhanced class activation maps and cross-domain attention mechanisms.

Across the four paradigms, several trends emerge: attention mechanisms (channel, spatial, and position variants) have become a de facto standard component of segmentation networks; lightweight design targets real-time embedded deployment; multi-task fusion jointly learns segmentation with severity estimation; and few-shot learning reduces annotation dependence. CNN encoder–decoder designs are the largest group of segmentation papers reviewed in this section. That is a count of papers. The numbers in Section 7 are not used to call one segmentation architecture the most accurate.

In the six comparison fields of Section 7, attention and edge modules target boundaries and low contrast, lightweight encoders target speed and memory, multi-task heads add severity to the mask, and few-shot or domain-adaptation models target scarce pixel labels. The aluminum-strip segmentation result is not used as a steel score. The NEU-Seg numbers remain reference only, so the highest recorded mIoU is not treated as the best segmentation architecture. Thin or elongated defects are the case addressed by edge and geometry modules, including DMS-UNet and the geometrically constrained convolution of Pu et al. Lightweight encoders are meant to reduce memory and latency, but the FPS values in Section 7 come from different devices, so they are not a speed ranking. What is still missing is a shared split and a shared account of how many pixel labels each method needs.

### 6.2. Method Based on Transformer

Convolutional Neural Networks (CNNs) excel at capturing local image features, but their limited receptive fields often fall short in steel defect segmentation tasks that require global semantic reasoning. This has created an urgent need for global contextual modeling capability. In this context, the Transformer architecture, with its powerful ability to capture global dependencies, has rapidly become a new paradigm in visual segmentation. Moreover, hybrid architectures combining CNN and Transformer achieve dual breakthroughs in accuracy and robustness by fusing local features and global representations. Swin–unet++ [144] introduces the Swin Transformer into the U-Net++ architecture, adding vice paths in the decoder to fuse multi-scale features. Sime et al. [145] use Transformer encoders to extract rich global features and combine them with CNN decoders for efficient upsampling and segmentation. F2P-Net [146] adopts a ViT+CNN dual-branch collaborative encoder (ViCNet), capturing global semantic features through a ViT backbone while lightweight CNN auxiliary branches enhance sensitivity to subtle defect textures, combined with multi-scale feature fusion and edge-enhanced visual cues. TransUNet [147] utilizes a Transformer to capture global contextual dependencies, combining U-Net’s decoder and skip connections to preserve local details. CRET [114] explicitly optimizes inter-class differences and intra-class consistency through class relationship enhancement modules (ICREM) and class token contrastive loss (CTC), and designs feature semantic balance bridge modules (FSBM) to fuse CNN’s local details with Transformer’s global semantics. Wu et al. [148] construct a unified region- and concept-level explainable AI framework (RC-XAI) based on CNN+Transformer hybrid architectures, enhancing model interpretability by combining region-level and concept-level explanations. Resformer-Unet [149] achieves efficient collaboration between local details and global semantics through parallel dual-branch encoders, combined with feature coupling modules to fuse multi-scale features.

In summary, Transformer-based segmentation approaches mainly appear as hybrid designs that pair global self-attention with CNN decoders and skip connections (Swin–UNet++, TransUNet, F2P-Net, Resformer-Unet), which improves global semantic consistency, while pure-Transformer solutions remain rare due to their data hunger and computational cost.

## 7. Literature-Reported Results and Discussion

This section consolidates the quantitative results reported for representative models on NEU-CLS, X-SDD, NEU-DET, GC10-DET and NEU-Seg, as introduced in Section 8. Table 9, Table 10, Table 11, Table 12 and Table 13 list the classification, detection and segmentation figures reported by the original papers together with the available split, input-resolution, pre-training, and augmentation details, so that readers can assess protocol comparability.

**Comparability disclaimer.** The figures in these tables are copied from the corresponding original publications, which differ in training/test splits, data augmentation, backbone pre-training, and hyperparameter settings. The rows are not a ranking: a lower figure does not mean an inferior architecture. Readers are encouraged to consult the original papers and reproduce the results under a unified protocol when a head-to-head comparison is required. This heterogeneity also motivates our call in Section 8 for protocol-aware benchmarking.

Each PDF was searched for how the data were divided. The source uses the word “random” for YOLOv8-BSPB (NEU-DET, training/validation 7:3, 1260/540, no test set), GSR-YOLO (both NEU-DET and GC10-DET, training/validation 9:1), DEW-YOLO (NEU-DET, 1440/180/180), and FDSNet (train/validation/test 6:2:2). Cross-validation is stated for LWFAG-LNet on NEU-CLS (80/20 and ten-fold), for MGJR (five-fold on all of its experiments), and for HiAM-Mamba (five-fold on both NEU-CLS and X-SDD). Other rows already show a ratio or image count where the PDF states one, but those PDFs do not call the split official, random, or cross-validation. No such split sentence was found for Fine-tuned CNN, SPEED, MMPA-Net, DDSNet, PGA-Net, GC-Net, IMPACT-Net, or for YOLO-SCM on NEU-DET and GC10-DET (YOLO-SCM states 7:2:1 only for its own BYGT-DET set). Those cells stay NR. No two rows are treated as directly comparable. A ratio such as 8:0:2 is train:validation:test. A count such as 1260/–/540 is a number of images. The X-SDD accuracies are those stated by LWFAG-LNet, MGJR, and HiAM-Mamba. The 94.11% figure cited by Shang et al. is not copied into the table.

On NEU-CLS, the same sources report LWFAG-LNet at 4.39 M parameters, 0.91 GFLOPs and 170 FPS for the compact setting that reaches 99.80% accuracy; MGJR at 21.14 M and 2.86 GFLOPs; and HiAM-Mamba at 21.25 M and 1.81 GFLOPs. Fine-tuned CNN and SDC-FS do not state parameter count or FPS next to the accuracies used above. Devices for these classification speeds are not stated, so the FPS figures are not compared.

**Observations across datasets.** On NEU-DET, the mAP@0.5 figures recorded in the detection table span 76.80% to 89.30%. Most of those entries are YOLO variants or YOLO-based hybrids. On GC10-DET, the figures for models developed for steel surface detection span 63.50% to 83.60%. The separate 86.20% entry is LFRSCNet, an aircraft-skin detector tested on GC10-DET; it is not grouped with the steel-designed models. GC10-DET images are 2048 × 1000, so a small defect occupies fewer pixels than in a 200 × 200 NEU-DET crop. On NEU-Seg, the recorded mIoU figures span 76.70% to 92.34%, and the listed models are mostly CNN encoder–decoders. These spans describe numbers printed by the source papers. They do not identify a best architecture.

Table 14 keeps the architecture-family grouping and places each literature-reported entry on its own row, so that FPS, parameter count, and FLOPs can be recorded per model. For families reviewed in Section 4, Section 5 and Section 6 (YOLO, DETR, hybrid CNN–Transformer, and CNN segmentation), the design discussion stays in those sections; models outside those sections are grouped under “Other”.

**Cross-method synthesis and selection guidance.** Table 15 organizes the reviewed approaches by the bottlenecks they address rather than by architecture family alone. Multi-scale fusion and detail preservation primarily address scale variation and spatial information loss, whereas attention enhancement and context modeling address feature ambiguity and background interference. These strategies are complementary: emphasizing defect-relevant features does not remove the need to preserve the spatial evidence of small defects. For elongated defects, morphology-aware designs such as WSO-YOLO11 [84] additionally emphasize directional structure, suggesting that generic multi-scale enhancement should be considered alongside defect geometry. For segmentation, boundary delineation requires particular attention to local detail, as illustrated by the edge-extraction design of DMS-UNet [119]. These examples support matching design choices to defect characteristics, but do not establish a universally superior strategy for any defect category.

The corresponding benefits should also be assessed against deployment and supervision costs. Additional feature-fusion paths or context-modeling modules may introduce computational overhead, whereas lightweight designs aim to reduce resource requirements. Table 14 provides quantitative reference points, including GSR-YOLO with 3.6 M parameters and 9.3 GFLOPs and FDSNet with 0.97 M parameters. Nevertheless, parameter counts and FLOPs alone do not establish practical latency, and the reported FPS values were obtained using different hardware and input settings. Training and inference costs should also be distinguished: the forward–reverse rectification method [52], for example, addresses few-shot prototype shift without additional inference cost. Consequently, reducing annotation requirements should not be equated with reducing computation, and few-shot methods should be evaluated under the intended label budget and adaptation protocol.

Taken together, the literature supports selecting strategies according to the dominant bottleneck: detail preservation for small or slender defects, feature discrimination and contextual reasoning for ambiguous backgrounds, efficient architectures for constrained deployment, and supervision-efficient learning for limited annotations. A stronger claim that a particular modification offers a favorable accuracy–cost trade-off requires within-study ablations or comparisons under matched experimental conditions. The heterogeneous results in Table 9, Table 10, Table 11, Table 12 and Table 13 therefore provide supporting evidence for this synthesis rather than a definitive ranking of architectural families.

## 8. Future Research Directions

Steel is the “food” of modern industry, and its surface quality directly determines the performance and lifespan of downstream high-end products such as automobiles, home appliances, and aerospace equipment. Over the past decade, deep learning methods—convolutional neural networks (CNNs), the YOLO series, and their many variants—have achieved remarkable results in specific scenarios. With the advancement of Industry 4.0 and intelligent manufacturing, however, existing technologies still face new challenges in complex scenarios such as high-resolution images, extreme data scarcity, superpixel segmentation, tiny and weak defects, and cross-modal quality assessment. In this section, we discuss the most promising research directions for addressing these challenges.

### 8.1. Standardized Benchmarking and Reproducibility

As pointed out in Section 7, the performance figures reported across the literature are currently difficult to compare directly because studies adopt different training/test splits, augmentation pipelines, and evaluation protocols, and the same metric (e.g., mAP@0.5) may be computed over different subsets. This problem has been recognized by earlier benchmarking efforts such as Chazhoor et al. [17]. Future work should therefore converge toward protocol-aware benchmarking: publishing exact data splits, reporting multiple complementary metrics (mAP@0.5 and mAP@0.5:0.95, FPS, parameter count), and releasing training configurations and code—the open-source resources compiled in Section 3.3 provide a starting point. A community-maintained leaderboard with fixed evaluation scripts would greatly accelerate fair method comparison and prevent the inflation of non-reproducible claims.

### 8.2. High-Resolution Steel Image Defect Detection

The core challenge in steel surface defect detection lies in the contradiction between high-resolution image processing efficiency and the precise identification of tiny defects. Production-line image resolutions often reach 2560 × 1600 pixels, which poses three major problems for mainstream detection models: excessive memory usage, dilution of small-target features during convolution, and loss of fine-grained textures.

An established strategy for high-resolution small-object detection is Slicing Aided Hyper Inference (SAHI) [178]. It divides an image into overlapping patches, performs detection on each patch, and merges the predictions in the original image coordinates. Full-image inference can also be incorporated to retain information about larger objects. For steel inspection, this provides a way to investigate small defects that may lose detail when an entire image is resized. However, patch size, overlap, boundary truncation, and prediction merging affect both detection quality and processing cost. Evaluation should therefore report these settings and measure latency or throughput per original image, including all slices and merging operations. Benefits demonstrated on other domains require separate validation on steel-defect data.

Beyond detection accuracy, precise measurement of defect dimensions and spatial distribution is also gaining attention, as demonstrated by YOLO-RH [179], which integrates detection with a practical defect measurement platform for automated industrial sorting.

### 8.3. Foundation-Model-Based Defect Segmentation

Vision foundation models such as SAM2 have achieved remarkable success across downstream visual tasks and are increasingly adopted in industrial perception systems. However, their generic decoders still struggle to handle multi-scale contextual cues and to preserve spatial details for small defect regions. Shen et al. [180] adapt SAM2 to seamless steel pipe internal surface defect detection through multi-scale guided cross-stage refinement, an early demonstration that foundation models, when equipped with domain-specific guidance, can reduce annotation dependence while retaining strong generalization. Future research should explore prompt-based zero-shot segmentation across steel grades, parameter-efficient fine-tuning for embedded deployment, and standardized benchmarks for measuring foundation-model transfer to defect datasets.

### 8.4. Few-Shot Steel Defect Detection

Few-shot steel defect detection has made steady progress around the challenge of identifying rare defect samples. Current mainstream methods focus on metric learning: prototype networks perform efficient nearest-neighbor classification by extracting class prototypes, and their improved versions maintain stable performance under extreme few-shot conditions by enhancing inter-class discrimination. Meta-learning techniques achieve rapid adaptation by combining with detection models, significantly shortening convergence cycles on new defect categories. In addition, synthetic data augmentation based on generative adversarial networks and diffusion models [181] improves sample diversity and enhances generalization. Nevertheless, clear limitations remain: few-shot detection performance still lags far behind fully supervised methods, and models become increasingly prone to confusion as the number of categories grows; cross-device and cross-steel-grade generalization is weak, with prominent domain shift; and non-uniform evaluation standards make method comparison difficult. Future directions include designing dynamic prototype evolution mechanisms to enhance feature discriminability, fusing prior knowledge from vision–language large models to improve cross-domain adaptability, and incorporating physical laws such as the rolling process to constrain the generation of synthetic data, thereby improving the metallurgical rationality of defect generation and the practical value of the models.

### 8.5. Small-Target Defect Detection

Small-target defect detection focuses on enhancing the visibility and discriminability of tiny defects. Current methods mainly refine feature representations with efficient attention mechanisms, reconstruct lightweight feature pyramids to fuse multi-scale information, and restore lost details through super-resolution. The field still faces significant challenges: pseudo-defects in industrial backgrounds cause high false positive rates; semantic gaps between features of different scales weaken fusion; and the added computational modules increase model burden, making it difficult to meet the stringent real-time requirements of production lines. Promising directions include adaptive enhancement mechanisms that embed industrial prior knowledge to suppress background interference, multi-scale feature fusion optimized through consistency regularization, and the co-design of algorithms and specialized hardware to achieve a step change in detection efficiency without sacrificing accuracy.

### 8.6. Superpixel Segmentation

The core idea of superpixel segmentation is to transform images from redundant pixel-grid representations into more compact, perceptually meaningful regional representations. In preliminary explorations of steel defect detection, researchers have applied Adaptive Simple Linear Iterative Clustering (ASLIC) to perform “superpixel over-segmentation” on steel defect images [182]. At present, performance evaluation of superpixel algorithms relies mainly on general computer vision datasets such as the Berkeley Segmentation Dataset (BSDS500) [183,184] and general metrics such as Boundary Recall (BR), Undersegmentation Error (UE), and Achievable Segmentation Accuracy (ASA) [183,184]. However, these datasets and metrics do not fully reflect the special requirements of steel defect detection, and the integration of superpixel segmentation with deep learning models remains awkward—data representation conflicts, fragmented processing pipelines, and the inability of end-to-end optimization. Future research needs to develop superpixel algorithms specialized for steel defect characteristics, construct differentiable superpixel layers for end-to-end co-optimization with deep learning, establish standardized evaluation benchmarks for defect detection, and explore the co-design of superpixels and lightweight models to improve accuracy, efficiency, and industrial applicability.

### 8.7. Steel Quality Assessment Combined with Large Language Models for Text Report Generation

Research on steel quality assessment and automatic report generation combined with large models is still in the exploratory stage. Existing methods attempt to align visual and textual features through multimodal models or rely on predefined templates to generate structured reports, preliminarily validating technical feasibility. Core challenges remain: general large models lack a deep understanding of steel defect terminology and mechanisms, leading to insufficiently professional generated content; high-quality image-report paired data are extremely scarce, constraining model performance; and general text evaluation metrics cannot measure the practical value of reports in industrial scenarios. Looking forward, research will shift toward domain-adaptive pre-training of vision–language large models to inject professional knowledge; multi-level “detection-description-diagnosis” generation architectures to reduce learning difficulty; and reinforcement learning to integrate industrial experts’ operational preferences and knowledge feedback into the optimization loop, ultimately generating high-quality analysis reports that are both accurate and actionable.

## 9. Conclusions

Steel surface defect detection has evolved from handcrafted-feature machine vision into a fully data-driven intelligent paradigm over the past decade. This survey cites 184 publications, 183 of them from 2015 to 2026, organized around the three core tasks of classification, detection, and segmentation, together with the supporting ecosystem of datasets, evaluation metrics, and open-source resources.

For classification, transfer learning with pre-trained backbones remains the workhorse for small-sample industrial scenarios, while lightweight designs, attention enhancement, and few-shot or weakly supervised strategies progressively address the constraints of limited annotations and embedded deployment. For detection, YOLO variants fill 7 of the 13 NEU-DET rows and 4 of the 13 GC10-DET rows in Section 7, which is 11 of 26 detection entries. The designs use multi-scale fusion, attention modules, lightweight backbones, and refined loss functions; meanwhile, DETR-based end-to-end frameworks, hybrid CNN–Transformer architectures, and emerging Mamba-based models provide complementary routes toward NMS-free pipelines and global context modeling. For segmentation, CNN encoder–decoder architectures such as U-Net variants remain the mainstream, with attention-enhanced, lightweight, multi-task, and few-shot branches enriching the design space, while Transformer-based and hybrid models steadily improve global semantic reasoning.

The literature-reported figures compiled in Section 7 span 76.80–89.30% mAP@0.5 on NEU-DET, 63.50–83.60% mAP@0.5 for the steel-designed GC10-DET entries, and 76.70–92.34% mIoU on NEU-Seg. LFRSCNet’s 86.20% on GC10-DET is a cross-domain test of an aircraft-skin model and is not included in that GC10 span. These ranges are copied from different papers. They are not a ranking. A later shared protocol would be required before the scores could be compared.

Limitations. Several limitations of this survey should be acknowledged. First, although the search covers the major bibliographic databases up to September 2026, non-indexed works, non-English publications, and the very latest online-first papers may still be underrepresented. Second, the survey focuses on image-based surface inspection; three-dimensional (point-cloud) representations and non-visual non-destructive testing modalities are out of scope. Third, the figures compiled in Section 7 are reproduced from the original publications. The sources do not use one training split or one evaluation protocol, and this survey does not rank the models. Fourth, taxonomy boundaries between architecture families are inherently fuzzy—many models combine CNN, Transformer, attention, and lightweight components—so our assignment of models to families reflects each paper’s primary design emphasis. Finally, the fast pace of this field means that new results published after the search cut-off date may not be reflected in the quantitative tables.

Despite the remarkable progress, fundamental challenges remain unsolved: defect sample scarcity and class imbalance, generalization across production lines and steel grades, small-target and weak-defect recognition under complex backgrounds, and the accuracy–speed trade-off on high-throughput lines. The most promising directions include high-resolution image processing with adaptive tiling and dynamic attention, few-shot and generative augmentation for rare defects, physics-guided and large-model-assisted quality assessment, and the co-design of algorithms with specialized industrial hardware.

In conclusion, deep learning has already reshaped steel surface defect detection and will continue to do so as models become lighter, benchmarks more standardized, and deployments more deeply integrated with manufacturing systems. We hope this survey serves as a practical map for both researchers entering the field and engineers building real production systems.

## Figures and Tables

**Table 1:** Full-text check of five earlier surveys. “No occurrence” means the stated terms do not appear in that PDF. A cited example is not the same as a comparison made by that survey.

Survey	Year	Models After 2023	Cross-Dataset Comparison	Accuracy with Efficiency	Deployment or Domain Shift
Luo et al. [15]	2020	No occurrence of 2024–2026, Mamba, DETR, or YOLOv10–v12	No occurrence of NEU-DET, GC10-DET, or X-SDD	Real-time inspection is a main theme; one cited result is 83 FPS. FLOPs do not occur	Industrial inspection systems are the subject. The word “deployment” does not occur
Wen et al. [16]	2022	No occurrence of 2024–2026, Mamba, or YOLOv10–v12	Lists GC10-DET and X-SDD. No comparison of one model across those sets	Method tables include FPS; some rows also give FLOPs and parameter counts	Section 2 is cameras and lighting. Real-time detection is discussed. One cited method uses domain adaptation
Chazhoor et al. [17]	2023	No occurrence of Mamba or YOLOv10–v12. Their models include deformable DETR	Own test: five models on NEU, five-fold CV, best 77.28%. GC10-DET and X-SDD appear only in related work	Accuracy is reported. FPS and FLOPs do not occur	Industrial use is stated in the abstract. No deployment section
Sohan et al. [9]	2024	YOLOv8 only. No occurrence of NEU-DET, GC10-DET, or X-SDD	Section 4.2 uses general detection sets, not steel sets	Section 4.2 defines FPS and FLOPs for YOLOv8	Section 5 and the pip package discuss general deployment, not steel lines
Shang et al. [18]	2026	Reviews strip-steel detectors through 2025, including YOLOv9, YOLOv10, and Vision Mamba	Groups published numbers on NEU-DET and GC10-DET, and states that cross-study comparison is not rigorous	Tables place mAP next to FPS, parameters, or GFLOPs	Lightweight deployment is a main theme. Domain generalization is discussed. No code list
**This survey**	2026	Section 4, Section 5 and Section 6 through 2026	Separate tables for NEU-CLS, X-SDD, NEU-DET, GC10-DET, and NEU-Seg	FPS, parameters, and FLOPs, with the device when the source states it	Section 1.1 states which non-steel papers may be included. Rows stay reference only

Bold marks the row for the present survey.

**Table 2:** Summary of classification datasets.

Datasets	Object	Numbers	Size
NEU-CLS http://faculty.neu.edu.cn/songkc/en/zdylm/263265 (accessed on 12 July 2026)	Hot-rolled steel strip	1800 images (6 defect types, 300 samples each)	200 × 200 pixels
NEU-CLS-64 http://faculty.neu.edu.cn/songkc/en/zdylm/263265 (accessed on 12 July 2026)	Hot-rolled steel strip	9 defect types	64 × 64 pixels
X-SDD [25] https://github.com/Fighter20092392/X-SDD-A-New-benchmark (accessed on 12 July 2026)	Hot-rolled steel strip	7 typical defects, 1360 defect images	640 × 640 pixels
SteelBlastQC https://www.selectdataset.com/dataset/82d51b3d89d46f589afda3aa1600de21 (accessed on 12 July 2026)	Sandblasted steel surface	1654 images	512 × 512 pixels
MSD-CLS https://github.com/successhaha/Gtnet (accessed on 12 July 2026)	Steel	500 images (10 defect types, 50 samples each)	224 × 224 pixels

**Table 3:** Summary of detection datasets.

Datasets	Object	Numbers	Size
NEU-DET http://faculty.neu.edu.cn/songkc/en/zdylm/263265 (accessed on 12 July 2026)	Hot-rolled steel strip	1800 images (6 defect types, 300 samples each)	200 × 200 pixels
GC10-DET https://github.com/lvxiaoming2019/GC10-DET-Metallic-Surface-Defect-Datasets (accessed on 12 July 2026)	Steel strip	3570 images with 10 defect types	2048 × 1000 pixels
Severstal-steel-defect https://www.kaggle.com/c/severstal-steel-defect-detection/data (accessed on 12 July 2026)	Steel strip	12,606 train + 5506 test images (4 defect types)	1600 × 256 pixels
MHPSDS https://github.com/clovermini/MVIT_metal_datasets (accessed on 12 July 2026)	Medium and Heavy Plate	4 typical defects, 680 images	256 × 256 pixels
Casting Billet Dataset https://github.com/clovermini/MVIT_metal_datasets (accessed on 12 July 2026)	Casting Billet	6 typical defects, 1060 images	96 × 106 to 3228 × 492
Steel Pipe Dataset https://github.com/clovermini/MVIT_metal_datasets (accessed on 12 July 2026)	Steel Pipe	4 typical defects, 1227 images	728 × 544 pixels

**Table 4:** Summary of segmentation datasets.

Datasets	Object	Numbers	Size
NEU_Seg http://faculty.neu.edu.cn/songkc/en/zdylm/263265 (accessed on 12 July 2026)	Hot-rolled steel strip	4470 images (3 defect types)	200 × 200 pixels
KolektorSDD2 https://modelscope.cn/datasets/OpenDataLab/KolektorSDD2 (accessed on 12 July 2026)	Multiple types	Training: 2085; Test: 1004	230 × 630 pixels
BSData https://hf-mirror.com/datasets/Gssmc/BSData (accessed on 12 July 2026)	Ball Screw	1104 three-channel images	multiple pixels
RSDDs dataset https://aistudio.baidu.com/dataset/detail/97732/file (accessed on 12 July 2026)	Rail surface	2 types (67 images; 128 images)	multiple pixels
SD-saliency-900 https://github.com/SongGuorong/MCITF/tree/master/SD-saliency-900 (accessed on 12 July 2026)	Bar Steel	900 images	200 × 200 pixels
FSSD-12 https://pan.baidu.com/s/15k5QQXX8Ha7yXNcdWQvV5Q?pwd=pfnc (accessed on 12 July 2026)	Steel strip	12 typical defects, 50 samples each	200 × 200 pixels
Severstal Steel Defect https://pan.baidu.com/s/1U9KIqbNx9F1Sv7MTH_PzXQ?pwd=ydc3 (accessed on 12 July 2026)	Steel strip	Training: 12,606; Test: 5506	1600 × 256 pixels

**Table 5:** Comparison of common evaluation metrics for different task types.

Task Type	Primary Metrics	Auxiliary Metrics (Industrial Concerns)
Classification	Accuracy, F1-score	ROC–AUC, False Detection Rate
Detection	mAP@0.5, mAP@0.5:0.95	FPS, Latency
Segmentation	IoU, Dice	BF-score, Model Parameter Quantity

**Table 6:** Summary of classification model codes.

Model Name	Year	Backbone Network	Keywords
SNN https://github.com/adipandas/one-shot-steel-surfaces (accessed on 12 July 2026)	2020	CNN	Computer vision; deep learning
ResNet50V2 https://github.com/shatibiswas/Fine-Tuned-Deep-Learning-model-for-Steel-Defect-Classification (accessed on 12 July 2026)	2022	CNN	Transfer learning; ResNet 50
FaNet https://github.com/VDT-2048/FSC-20 (accessed on 12 July 2026)	2023	ResNet12	Feature-aware; feature-attention convolution
SteelNet https://github.com/musickevan1/steelnet (accessed on 12 July 2026)	2023	ResNet18	ResNet 18; transfer learning
SuperSimpleNet https://github.com/blaz-r/SuperSimpleNet (accessed on 12 July 2026)	2024	CNN	Supervised learning; unsupervised learning
ViT-MTL https://github.com/toqaalaa20/Metal-surface-defects-detection?tab=readme-ov-file (accessed on 12 July 2026)	2024	ViT; CNN	Self-attention mechanism; multi-layer perceptrons
LoHi-WELD https://github.com/SylvioBlock/LoHi-Weld (accessed on 12 July 2026)	2024	CSPDarknet	Deep learning

**Table 7:** Summary of detection model codes.

Model Name	Year	Backbone Network	Keywords
AGCA https://github.com/C1nDeRainBo0M/AGCA (accessed on 12 July 2026)	2023	YOLO	Adaptive graph convolution; channel attention
GC-Net https://github.com/Ghlerrix/GC-Net (accessed on 12 July 2026)	2025	YOLOv8	Global attention; cascade fusion network
GLNet https://github.com/sod76/GLNet (accessed on 12 July 2026)	2025	VMamba; CNN (ResNet50)	Global–local fusion; CPOE; SSCA attention
CAT-YOLO https://github.com/lzxinxinxin/CAT-YOLO (accessed on 12 July 2026)	2024	YOLOv5s	Adaptive fusion; decoupling head
TAFENet https://github.com/moonlike0622/a-two-stage-attention-based-feature-enhancement-network-for-strip-steel-surface-defect-detection (accessed on 12 July 2026)	2024	NextViT	Attention mechanism; feature fusion

**Table 8:** Summary of segmentation model codes.

Model Name	Year	Backbone Network	Keywords
CPANet https://github.com/VDT-2048/CPANet (accessed on 12 July 2026)	2023	ResNet-50	Few-shot learning; cross position aggregation network
SME-DeepLabV3+ https://github.com/Eric-863/LGASeg (accessed on 12 July 2026)	2025	StarNet	Multi-scale attention; star operation

**Table 9:** Literature-reported classification results and experimental settings on NEU-CLS. Params, FLOPs and FPS are taken from the same source as the accuracy. NR means that source does not state the item. The device for the 170 FPS is not stated. The rows are not directly comparable.

Model (Year)	Split Ratio (Tr:V:Te)	Input	Pre-Training	Augmentation	Accuracy (%)	Params	FLOPs (G)	FPS
Fine-tuned CNN [150] (2022)	NR	200 × 200	ImageNet	Flip, rotation, translation, scaling, DCGAN	99.11	NR	NR	NR
SDC-FS [151] (2023)	1:0:59 (5-shot)	224 × 224	ImageNet	NR	97.69 ± 0.32	NR	NR	NR
LWFAG-LNet [37] (2025)	8:0:2, 10-fold	224 × 224	NR	NR	99.80	4.39 M	0.91	170
MGJR [152] (2025)	5-fold	224 × 224	Teacher pre-trained (dataset NR)	Crop, rotation, flip, AGTS	99.98	21.14 M	2.86	NR
HiAM-Mamba [153] (2026)	5-fold	224 × 224	NR	Flip, rotation, ColorJitter	99.90 ± 0.10	21.25 M	1.81	NR

**Table 10:** Literature-reported classification results on X-SDD. These three papers state an X-SDD accuracy together with the fields below. The rows are reference only.

Model (Year)	Split	Input	Params	FLOPs (G)	Accuracy (%)
LWFAG-LNet [37] (2025)	NR	NR	4.39 M	0.91	98.49
MGJR [152] (2025)	NR	NR	21.14 M	2.86	99.64
HiAM-Mamba [153] (2026)	5-fold	224 × 224	21.25 M	1.81	99.15 ± 0.11

**Table 11:** Literature-reported results and experimental protocols on NEU-DET. The results were obtained under heterogeneous settings and are not directly comparable.

Model (Year)	Split Ratio (Tr:V:Te)	Input	Pre-Training	Augmentation	Reported Result (mAP@0.5)
SDDNet [154] (2021)	7:0:3	512 × 512	ImageNet	Flip, crop	88.80%
FANet [155] (2022)	7:0:3	400 × 400	ImageNet	Flip, crop, photometric distortion, expansion	89.30%
YOLOv5-ISC [156] (2023)	6:2:2	448 × 448	NR	NR	88.30%
RDD-YOLO [67] (2023)	8:0:2	416 × 416	NR	Mosaic	81.10%
DEW-YOLO [157] (2024)	8:1:1, random	640 × 640	NR	NR	80.30%
YOLOv8-BSPB [158] (2024)	7:3:0, random	640 × 640	NR	NR	86.90%
TD-Net [159] (2024)	7:0:3	640 × 640	None	NR	76.80%
SDDF-Net [160] (2024)	8:0:2	416 × 416	NR	NR	78.80%
PCT-Net [81] (2025)	8:0:2	224 × 224	COCO	Mosaic, flip, translation	80.70%
GSR-YOLO [92] (2025)	9:1:0, random	640 × 640	NR	CLAHE	84.70%
MPA-YOLO [74] (2025)	7:1.5:1.5	640 × 640	NR	NR	81.50%
SA-FPN [161] (2025)	1260/–/540	200 × 200	ImageNet	NR	86.80%
YOLO-SCM [162] (2026)	NR	640 × 640	NR	Mosaic, flip, color	80.70%

**Table 12:** Literature-reported results and experimental protocols on GC10-DET. The results were obtained under heterogeneous settings and are not directly comparable.

Model (Year)	Split Ratio (Tr:V:Te)	Input	Pre-Training	Augmentation	Reported Result (mAP@0.5)
LAACNet [163] (2024)	8:1:1	NR	NR	NR	66.80%
GSR-YOLO [92] (2025)	9:1:0, random	544 × 544	NR	CLAHE	79.40%
PCT-Net [81] (2025)	8:0:2	224 × 224	COCO	NR	69.20%
SA-FPN [161] (2025)	8:1:1	512 × 250	ImageNet	NR	74.40%
LCRAA-Net [164] (2025)	8:1:1	1024 × 500	NR	NR	74.50%
ELA-YOLO [165] (2025)	8:0:2	640 × 640	NR	Contrast adjustment, sharpening	74.30%
FC-DETR [166] (2025)	8:1:1	NR	ImageNet-1K	NR	83.60%
ARYOLO [78] (2025)	8:2:0	320 × 320	NR	Mosaic	71.20%
GC-Net [167] (2025)	NR	NR	NR	Oversampling, small object aug.	63.50%
IMPACT-Net [168] (2026)	NR	640 × 640	NR	HSV, translation, scaling, flip, random erasing, Mosaic	71.40%
CFC-CenterNet [169] (2026)	8:1:1	NR	None	Random crop, random scale	69.40%
LFRSCNet [170] (2026; aircraft-skin model, cross-domain)	8:1:1	NR	NR	NR	86.20%
YOLO-SCM [162] (2026)	NR	NR	NR	NR	71.15%

**Table 13:** Literature-reported results and experimental protocols on NEU-Seg. The results were obtained under heterogeneous settings and are not directly comparable.

Model (Year)	Split Ratio (Tr:V:Te)	Input	Pre-Training	Augmentation	Reported Result (mIoU)
PGA-Net [171] (2019)	3630/–/840	192 × 192	ImageNet	Rotation, Random crop, Horizontal reflection	82.15%
FDSNet [172] (2022)	6:2:2, random	200 × 200	NR	Scale (0.5–2.0), Crop 450 × 450, Flip	78.80%
SPEED [173] (2023)	NR	448 × 448	NR	Random horizontal flipping, Rotation	92.34%
MMPA-Net [174] (2024)	3600/–/900	224 × 224	ImageNet	Horizontal flip, Vertical flip, Horizontal-vertical flip, Rotation	86.62%
DDSNet [11] (2024)	NR	200 × 200	NR	Flip, scale, crop, photometric distortion	85.12%
LDM [175] (2024)	2541/1089/840	200 × 200	NR	Flip, Gaussian noise, CLAHE, Brightness, Gamma, Sharpening, Blurring	76.70%
WFF-Net [128] (2025)	7:1:2	256 × 256	ResNet-50	Horizontal flip, Vertical flip, Color dithering	85.70%
PFENet [176] (2025)	7:0:3	224 × 224	ImageNet	rotation, horizontal-vertical flipping, scaling	77.00%
EA-SegNet [177] (2025)	2178/726/840	200 × 200	NR	NR	85.20%

**Table 14:** Per-model literature-reported entries grouped by architecture family. Detection entries report mAP@0.5; NEU-Seg entries report mIoU. A dash marks a metric that does not apply to that task. FPS, parameter count, and FLOPs are transcribed from the source papers. NR marks a quantity the source does not report as an absolute value. Hardware differs by paper, so the FPS column is not comparable across rows. Stated devices include an RTX 3090 for YOLOv8-BSPB, an RTX 4060 Ti for GSR-YOLO, an RTX 3080 for PCT-Net, and an RTX 4090 for LFRSCNet. LFRSCNet’s 7.16 M and 65.3 FPS are its GC10-DET cross-domain figures. YOLO-SCM reports 62 FPS on an RTX 4080 for its own BYGT-DET experiment, not for the NEU-DET or GC10-DET generalization scores, so those two rows leave FPS as NR. Its absolute parameter count and FLOPs are not stated.

Family (Reviewed in)	Model (Dataset)	mAP@0.5	mIoU	FPS	Params	FLOPs (G)
YOLO series and variants (Section 5.1)	YOLOv5-ISC (NEU-DET)	88.30%	–	25	NR	NR
	RDD-YOLO (NEU-DET)	81.10%	–	57.8	NR	NR
	DEW-YOLO (NEU-DET)	80.30%	–	91	7.45 M	11.0
	YOLOv8-BSPB (NEU-DET)	86.90%	–	103.1	5.5 M	10.4
	MPA-YOLO (NEU-DET)	81.50%	–	97.1	12.1 M	34.9
	ELA-YOLO (GC10-DET)	74.30%	–	101.3	5.4 M	16.5
	GSR-YOLO (NEU-DET)	84.70%	–	154	3.6 M	9.3
	GSR-YOLO (GC10-DET)	79.40%	–	32	3.6 M	9.3
	YOLO-SCM (NEU-DET)	80.70%	–	NR	NR	NR
	YOLO-SCM (GC10-DET)	71.15%	–	NR	NR	NR
DETR-based (Section 5.2)	FC-DETR (GC10-DET)	83.60%	–	NR	56.5 M	143
Hybrid CNN–Transformer (Section 5.5)	PCT-Net (NEU-DET)	80.70%	–	51.1	10.3 M	24.3
	PCT-Net (GC10-DET)	69.20%	–	51.1	10.3 M	24.3
	ARYOLO (GC10-DET)	71.20%	–	140	NR	52
CNN-based segmentation (Section 6.1)	PGA-Net (NEU-Seg)	–	82.15%	41–49	NR	NR
	FDSNet (NEU-Seg)	–	78.80%	186.1	0.97 M	NR
	SPEED (NEU-Seg)	–	92.34%	58.38	NR	NR
	MMPA-Net (NEU-Seg)	–	86.62%	NR	NR	NR
	DDSNet (NEU-Seg)	–	85.12%	NR	NR	0.97
	LDM (NEU-Seg)	–	76.70%	NR	NR	NR
	WFF-Net (NEU-Seg)	–	85.70%	NR	NR	71.14
	PFENet (NEU-Seg)	–	77.00%	35.74	NR	NR
	EA-SegNet (NEU-Seg)	–	85.20%	69.44	27.35 M	NR
Other	SDDNet (NEU-DET)	88.80%	–	31.0	NR	NR
	FANet (NEU-DET)	89.30%	–	34	35.9 M	NR
	TD-Net (NEU-DET)	76.80%	–	NR	7.06 M	NR
	SDDF-Net (NEU-DET)	78.80%	–	NR	NR	NR
	LAACNet (GC10-DET)	66.80%	–	43	6.10 M	NR
	SA-FPN (NEU-DET)	86.80%	–	51	63.23 M	NR
	LCRAA-Net (GC10-DET)	74.50%	–	122.3	9.16 M	22.93
	GC-Net (GC10-DET)	63.50%	–	38	NR	NR
	IMPACT-Net (GC10-DET)	71.40%	–	400	NR	NR
	CFC-CenterNet (GC10-DET)	69.40%	–	30.7	25.9 M	109.3
	LFRSCNet (GC10-DET, cross-domain)	86.20%	–	65.3	7.16 M	NR

**Table 15:** Cross-method synthesis of representative strategies for steel surface defect inspection.

Methodological Strategy	Primary Objective	Typical Benefits	Computational Implications	Applicable Defects or Conditions	Main Limitations
**Multi-scale fusion and detail preservation** MSFT-YOLO [65]; SFW-YOLO [75]	Address scale variation and loss of spatial detail	Combine shallow localization cues with deep semantics; preserve small-defect features	Additional fusion paths and high-resolution features may increase computation and memory use	Small defects and images containing defects at substantially different scales	Cross-scale semantic mismatch can weaken fusion; effectiveness remains dependent on available image detail
**Attention enhancement and context modeling** CBAM-YOLOv5 [86]; TransUNet [147]	Reduce background interference and strengthen contextual representation	Emphasize defect-relevant features and combine local information with broader context	Cost depends on the mechanism; channel/spatial reweighting and global self-attention have different computational requirements	Complex textures, ambiguous defect–background appearance, and defects requiring contextual interpretation	Context enhancement alone does not ensure preservation of fine details; additional complexity can constrain deployment
**Lightweight architecture design** GSR-YOLO [92]; DSUNet [131]	Meet memory, computation, and latency constraints	Reduce model size or arithmetic complexity through efficient modules and convolutions	Designed to reduce parameters or FLOPs; actual latency remains hardware- and implementation-dependent	High-speed production lines and resource-constrained devices	Fine-detail retention requires validation; reduced theoretical complexity does not establish deployment speed
**Learning with scarce annotations** Forward–reverse rectification [52]; CPANet [23]	Reduce dependence on extensive labeled data	Support classification or segmentation with limited annotated examples	Annotation savings do not necessarily reduce training or inference costs; the method in [52] reports no additional inference cost	Rare defect categories and settings with few labeled samples or expensive pixel annotations	Dependence on the few-shot protocol and reference samples; uncertain generalization across acquisition conditions

Bold marks the strategy name in each row. The methods listed beneath it are examples.

## Data Availability

No new data were created or analyzed in this study. Data sharing is not applicable to this article.

## Abbreviations

The following abbreviations are used in this manuscript:

AP	Average Precision
AUC	Area Under the Curve
BiFPN	Bidirectional Feature Pyramid Network
CNN	Convolutional Neural Network
DETR	DEtection TRansformer
FN	False Negative
FP	False Positive
FPS	Frames Per Second
FPN	Feature Pyramid Network
IoU	Intersection over Union
MAE	Mean Absolute Error
mAP	mean Average Precision
mIoU	mean Intersection over Union
MSE	Mean Squared Error
NMS	Non-Maximum Suppression
PA	Pixel Accuracy
ROC	Receiver Operating Characteristic
SA	Spatial Attention
SAM2	Segment Anything Model 2
SSM	State Space Model
TN	True Negative
TP	True Positive
ViT	Vision Transformer
YOLO	You Only Look Once
